# Acoustic and visual cetacean surveys reveal year-round spatial and temporal distributions for multiple species in northern British Columbia, Canada

**DOI:** 10.1038/s41598-022-22069-4

**Published:** 2022-11-10

**Authors:** Héloïse Frouin-Mouy, Xavier Mouy, James Pilkington, Elizabeth Küsel, Linda Nichol, Thomas Doniol-Valcroze, Lynn Lee

**Affiliations:** 1 JASCO Applied Sciences Ltd, Victoria, BC Canada; 2 Fisheries and Oceans Canada, Cetacean Research Program, Pacific Biological Station, Nanaimo, BC Canada; 3 JASCO Applied Sciences Inc, Vancouver, WA USA; 4 Gwaii Haanas National Park Reserve, National Marine Conservation Area Reserve, and Haida Heritage Site, Skidegate, BC Canada; 5 Present Address: University Corporation for Atmospheric Research (UCAR|CPAESS), under contract to National Oceanic and Atmospheric Administration, National Marine Fisheries Service, Southeast Fisheries Science Center, Miami, FL USA; 6 Present Address: Integrated Statistics, Inc., Under Contract to National Oceanic and Atmospheric Administration, National Marine Fisheries Service, Northeast Fisheries Science Center, Woods Hole, MA USA

**Keywords:** Marine biology, Animal migration, Ecology

## Abstract

Cetaceans spend most of their time below the surface of the sea, highlighting the importance of passive acoustic monitoring as a tool to facilitate understanding and mapping their year-round spatial and temporal distributions. To increase our limited knowledge of cetacean acoustic detection patterns for the east and west coasts of Gwaii Haanas, a remote protected area on Haida Gwaii, BC, Canada, acoustic datasets recorded off SG̱ang Gwaay (Sep 2009–May 2011), Gowgaia Slope (Jul 2017–Jul 2019), and Ramsay Island (Aug 2018–Aug 2019) were analyzed. Comparing overlapping periods of visual surveys and acoustic monitoring confirmed presence of 12 cetacean species/species groups within the study region. Seasonal patterns were identified for blue, fin, humpback, grey and sperm whale acoustic signals. Killer whale and delphinid acoustic signals occurred year-round on both coasts of Haida Gwaii and showed strong diel variation. Cuvier’s, Baird’s, beaked whale and porpoise clicks, were identified in high-frequency recordings on the west coast. Correlations between environmental factors, chlorophyll-a and sea surface temperature, and cetacean acoustic occurrence off Gwaii Haanas were also examined. This study is the first to acoustically monitor Gwaii Haanas waters for an extended continuous period and therefore serves as a baseline from which to monitor future changes.

## Introduction

Monitoring for the presence of cetaceans is challenging as they generally spend a small proportion of their time at the surface where they can be observed. This challenge is particularly acute in remote places where few people live and access by researchers for visual surveys is limited by ship-time, funding, weather and sea state. Gwaii Haanas is a protected area that encompasses the southern third of Haida Gwaii (HG), a remote archipelago off the north coast of British Columbia (BC; Canada). Previous research identified the southeastern area HG as the most species-rich habitat for marine mammals BC^1^. Nevertheless, movements and distributions of cetaceans off southwestern HG remain poorly understood due to the many challenges of offshore visual monitoring, particularly in winter.

Marine mammals are important biological contributors to underwater soundscapes, and cetaceans in particular rely almost exclusively on sound for communication, navigation, mating and foraging^2^. Given that most marine mammals produce species-specific sounds underwater, passive acoustic monitoring (PAM) can detect the presence of multiple cetacean species in remote environments over long timeframes and large areas. Importantly, PAM is a non-intrusive and cost-effective complementary approach to visual surveys and satellite transmitter tracking for monitoring cetacean temporal and spatial distribution and habitat use (e.g.,^3^). Acoustic monitoring is less restricted by weather and sea state conditions than visual surveys and is unaffected by visibility. To date, existing comparisons of concurrent visual and PAM datasets in BC have not described cetacean distributions or occurrence across space and time (e.g.,^4^) or focus only on one species (e.g.,^5^).

The behaviour and ecology of cetaceans (e.g., foraging, distribution, migration) is influenced by abiotic (physiographic and dynamic oceanographic variables; see^6^ for a review) and biotic factors (e.g., food availability; ^7,8^). Remotely-sensed environmental parameters such as satellite-derived surface chlorophyll-a (chl-a) concentrations (index of phytoplankton biomass as proxy for primary productivity^9^) and sea surface temperature (SST) provide environmental context for conditions that influence cetacean habitat use, and can potentially identify biological hotspots for cetaceans^10,11^.

Here, PAM data from the Gwaii Haanas marine monitoring program and visual cetacean surveys conducted by Fisheries and Oceans Canada (DFO) were used to: (1) document cetacean presence off the east and west coasts of Gwaii Haanas; (2) compare monitoring results from acoustic and visual surveys; (3) determine seasonal and diel acoustic occurrence by species and location; (4) investigate the correlations between environmental factors (chl-a and SST) and cetacean acoustic occurrence; and (5) explore contributions of cetacean calls to the underwater soundscape off the west coast of Gwaii Haanas. This study highlights the critical role of collaborations among organizations and experts required to implement such cetacean monitoring work, particularly in remote and rugged coastal regions.

## Methods

### Study area

**Figure 1:**
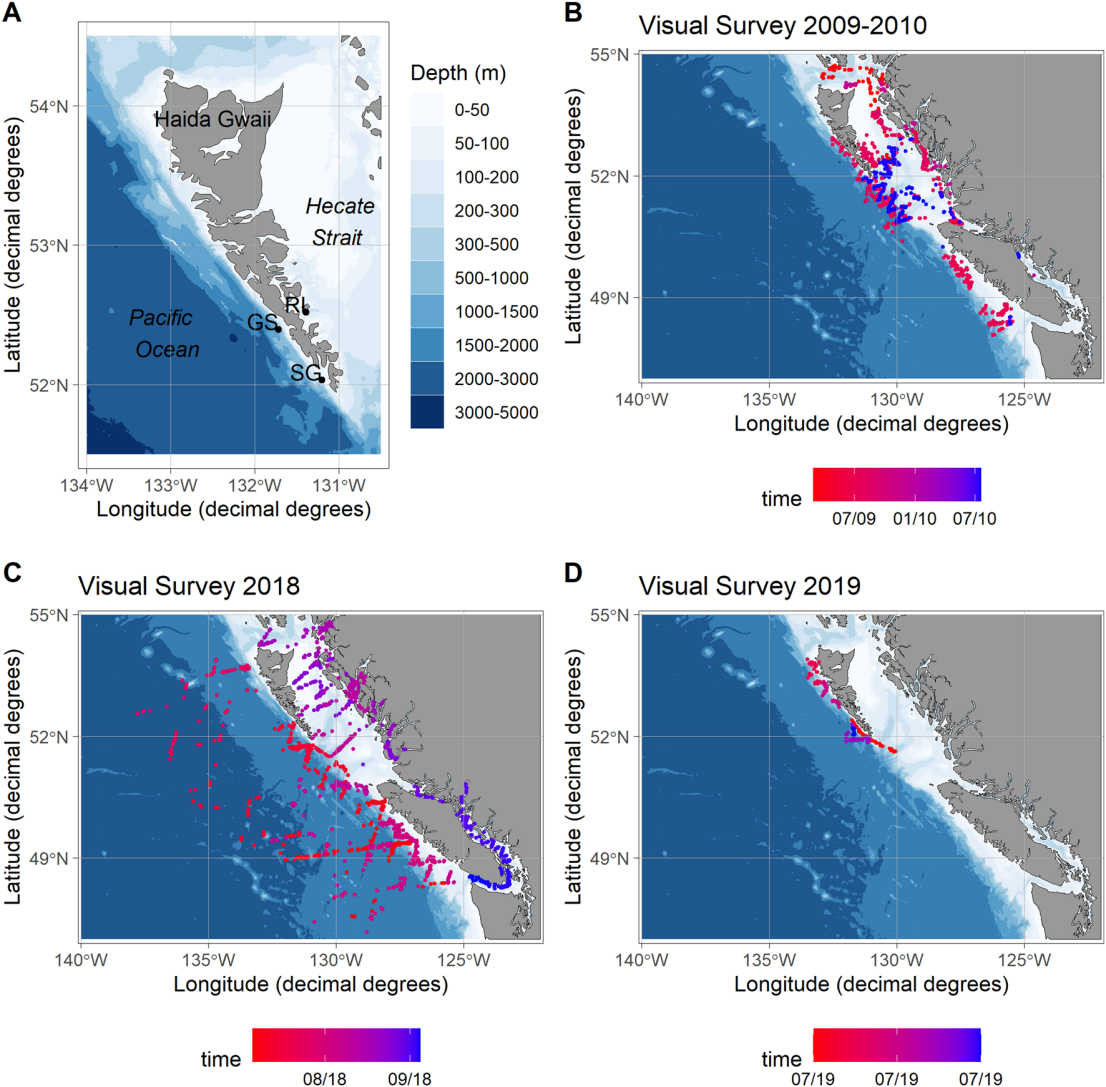
(**A**) Map of Haida Gwaii, BC, Canada, showing the three hydrophone locations (black dots)—Gowgaia Slope (GS, 2017–2019), SG̱ang Gwaay (SG, 2009–2011) and Ramsay Island (RI, 2018–2019). (**B**–**D**) Maps showing location and timing (month/year) of cetaceans sightings that overlapped with acoustic recording periods (**B**: 2009–2010; **C**: 2018; and **D**: 2019). Maps were created using the ggOceanMaps package (version 1.2.6) in R^122^.

Gwaii Haanas National Park Reserve, National Marine Conservation Area Reserve, and Haida Heritage Site (Gwaii Haanas) is located in southern HG, an archipelago in northwestern BC, Canada, on the southeastern extremity of the Alaska Current Large Marine Ecosystem in the Gulf of Alaska, USA (Fig. 1). Known for its diverse ecosystems, distinct and rich flora and fauna, and Haida culture^12^, Gwaii Haanas is one of seven national park reserves and the only national marine conservation area reserve in BC. Gwaii Haanas encompasses 5000 km^2^ of land and sea that is cooperatively managed by the Council of the Haida Nation and the Government of Canada as represented by Parks Canada (PC) and DFO, being the only area protected from mountain top to seafloor in western Canada. Twenty-four cetacean species are known to frequent Gwaii Haanas waters ^13^, including seven mysticetes and 17 odontocetes (Table S1).

### Passive acoustic monitoring

Underwater sound was recorded at three locations around Gwaii Haanas: Gowgaia Slope (GS, ~ 740 m depth) on the west coast, off SG̱ang Gwaay (SG, ~ 100 m depth) on the southwest coast, and off Ramsay Island (RI, ~ 150 m depth) on the east coast (Fig. 1) at different time periods between 2009 and 2019 (Table 1; SG: 2009–2011; GS: 2017–2019; RI: 2018–2019). There was no overlap between detection ranges of GS and RI because the recorders were located on different sides of the island. Each site used a different acoustic recorder: Autonomous Underwater Recorder for Acoustic Listening (AURAL-M2; Multi-Electronique) at SG, Autonomous Multichannel Acoustic Recorder (AMAR G3; JASCO Applied Sciences) at GS, and Song Meter (SM2M; Wildlife Acoustics) at RI (Table 1). Hydrophone deployments and retrievals were conducted as a collaboration between Gwaii Haanas, DFO Ocean Sciences Division, and DFO Cetacean Research Program (CRP). Acoustic analyses were conducted by JASCO Applied Sciences.

**Table 1:** Location, depth, acoustic recorder type, and operation time periods of the analyzed datasets.

Location	Depth (m)	Latitude	Longitude	Recorder	Start (D/M/Y)	End (D/M/Y)	Duration (days)	Duty cycle
SG̱ang Gwaay (SG)	100	52.033617	− 131.2009	AURAL M2	20/09/09	15/07/10	298	7 min at 16,384 Hz 23 min off
SG̱ang Gwaay (SG)	98	52.033483	− 131.2009	AURAL M2	15/07/10	17/05/11	306	9 min at 16,384 Hz 21 min off
Gowgaia Slope (GS)	743	52.39355	− 131.7130	AMAR G3	12/07/17	11/07/18	368	5 min 41 s at 16 kHz 1 min 4 s at 250 kHz 8 min 15 s off
Gowgaia Slope (GS)	741	52.39362	− 131.7132	AMAR G3	11/07/18	8/07/19	363	5 min 41 s at 16 kHz 1 min 4 s at 250 kHz 8 min 15 s off
Ramsay Island (RI)	150	52.51783	− 131.3863	SM2M	22/08/18	5/08/19	349	5 min at 96 kHz 55 min off

Recorders at each site varied in recording durations, schedules, and sample frequencies (Table 1). Recorders were deployed to seafloor depths between 200 and 741 m as a vertical mooring with the hydrophone approximately 10 m above the seafloor for GI and SG, and approximately 50–100 m above the seafloor for RI. AURALs were fitted with an HTI-96-MIN omnidirectional hydrophone (High Tech Inc., nominal sensitivity: − 164 dB re 1 V/μPa), had an analog gain of 16 dB and were set to record at a sampling frequency of 16,384 Hz with a resolution of 16-bit. AMARs were fitted with a GTI M36-V35-100 omnidirectional hydrophone (GeoSpectrum, Inc., nominal sensitivity: − 165 ± 3 dB re 1 V/μPa) and were set to record at two different sampling frequencies. The low-frequency (16 kHz) and high frequency (250 kHz) recording channels had a resolution of 24-bit and 16-bit, respectively, and an analog gain of 6 dB and 0 dB, respectively. The SM2M was fitted with an HTI-92-WB hydrophone (Wildlife Acoustics’ ‘low-noise’ hydrophone option), set to sample at a frequency of 96 kHz on 16-bit and with an analog gain of 0 dB.

#### Ambient sound level analyses

Acoustic analyses were corrected for calibrated system response and quantitatively described the underwater soundscape recorded for each dataset. The raw pressure waveform data were scaled according to the mean calibrated voltage sensitivity of the recorders and adjusted for the amplitude and frequency response of the hydrophone sensor. An end-to-end calibration of the AMAR was performed from about 4 Hz to 1000 Hz on both channels before the first and second deployments. Additionally, the AMAR was calibrated before and after each deployment with a pistonphone type 42AC precision sound source (G.R.A.S. Sound & Vibration A/S) at 250 Hz. The AURALs were not calibrated in the field. Therefore, the recorder sensitivity was estimated using the mean sensitivity from 39 of JASCO’s previously deployed and calibrated AURAL-M2 recorders^14^. The SM2M was calibrated before the deployment in the field (an end-to-end calibration was performed).

Analyses were based on 1-min average power spectral density of the data computed from fast Fourier transforms (FFTs) of 1 s of data overlapped by 0.5 s (120 averages). Acoustic metrics used to quantify the ambient sound were: (1) root-mean-square sound pressure level (rms SPL) and (2) power spectral density (PSD) level.

#### Cetacean call detections

To detect cetacean calls, a combination of automated detectors and manual analysis by expert analysts was used (Table S1). Automated detectors identified acoustic signals potentially produced by cetaceans. Automated detections were manually reviewed (validated) within a sample of each data set (Table S2), and results of detectors were also validated (details in Supplementary Sect. 1.1). The performance of automatic detectors was generally high but varied between species and locations (Table S3).

To examine seasonal and diel patterns of cetacean acoustic occurrence, both automated and manually validated datasets were selected for analysis. All data were binned by hour and results plotted by day. The Astral python package v2.2 (https://astral.readthedocs.io) was used to extract sunrise/sunset times based on the NOAA Solar Calculator (https://gml.noaa.gov/grad/solcalc/calcdetails.html). ‘Light’ periods were defined as those hours between sunrise and sunset, and ‘dark’ hours were between sunset and sunrise.

### Acoustic detection ranges

Species-specific acoustic detection ranges of cetacean vocalizations were estimated by calculating the distance from an acoustic recorder where the received sound level (RL in dB re 1 µPa) of a vocalization was higher than a detection threshold (DT) above the ambient noise level in the same frequency band. These distances can be greater than the distances at which conspecifics can detect each other because marine mammal hearing is often not as sensitive as acoustic recorders. RL of a cetacean vocalization at the location of the acoustic recorder is the difference between the source level (SL in dB re 1 µPa at 1 m) (i.e., a cetacean) and propagation loss (PL in dB re 1 m), representing the reduction of sound amplitude as it propagates from the cetacean to the hydrophone^15^:

1 $${\text{RL }} = {\text{ SL}} - {\text{PL}}$$

PL was calculated using the specialized acoustic models RAM^16^ and Bellhop^17^ that account for source and receiver depths, and environmental and bathymetric conditions between source and receiver. For this analysis, cetacean vocalization SLs (Supplementary Sect. 2.1) and source depth (Supplementary Sect. 2.2) were obtained from published literature, and ambient noise levels (NLs) recorded at GS and RI (Supplementary Sect. 2.3). PL was modelled for two sites along four radials in different directions to sample PL characteristics as a function of range and azimuth (Supplementary Sect. 2.4).

The maximum distance a vocalization can be detected is that at which the vocalization’s RL exceeds NL at the recorder in the same frequency band by at least the DT:

2 $$RL\left(f,z,r\right)\ge NL\left(f\right)+DT$$

For frequency (*f*) in Hertz, depth (*z*) in metres, and range (*r*) in metres. NL varied substantially over time due to dynamic sounds from many sources, including passing ships, wind, and breaking waves, and often from non-acoustic flow noise caused by tidal currents. Ambient NLs are required in Eq. (2) for estimating detection ranges of vocalizations for different species. Sound pressure levels (SPLs) from two months of consecutive recordings at GS and RI were used to represent noise levels for summer at each monitoring location. For GS, the 250 kHz AMAR sampling channel was used to cover vocalization frequencies from all species of interest. SPLs for NL estimates at both locations were calculated and reported separately for each frequency band defined in Table S4.

DT was set to 0 dB for this analysis, as automated detectors typically perform well above that signal-to-noise ratio SNR^18^. The DT used here strictly represents the signal processing DT for automated detectors and is not related to the listening DT of the animals.

At a given source depth, the detection range was estimated separately for each frequency band of the vocalization (Supplementary Section 2.1), and the final detection range was defined as:

3 $${R}_{max}=arg \; {max}_{f} \left(R\left(f\right)\right)$$

where *R(f)* is the detection range at frequency band *f.* The bandwidth of the selected frequency bands varied for each type of cetacean vocalization and was assumed to be the smallest bandwidth necessary for an automated detector to detect that type of vocalization (Table S4). Frequency boundaries also varied for each cetacean vocalization type and were chosen to cover the full frequency range of each vocalization (Table S4).

Detection range was calculated for each minute of ambient noise data from the AMAR recordings. To estimate detection range of cetacean vocalizations, a Monte Carlo simulation was used to account for measured variability in SLs and animal depths. Detection ranges were calculated 10,000 times for all NLs available by randomly choosing 100 normally distributed SL values, with the means and standard deviations (described in Supplementary Section 2.1), and 100 animal depths selected from vocalization depth distributions defined from DFO animal tagging data or scientific literature (Supplementary Section 2.2). Each iteration of the Monte Carlo process provided a probability of detection by the 10th, 50th, and 90th percentiles at each range from the hydrophone.

Detection ranges were estimated for vocalizations from five species at GS—northern resident killer whale (*Orcinus orca*), fin whale (*Balaenoptera physalus*), blue whale (*Balaenoptera musculus*), humpback whale (*Megaptera novaeangliae*), and sperm whale (*Physeter macrocephalus*)—and from five species at RI—northern resident killer whale, fin whale, humpback whale, gray whale (*Eschrichtius robustus*), and Pacific white-sided dolphin (*Lagenorhynchus obliquidens*). Sound propagation can vary spatially and temporally; therefore, detection ranges were estimated independently along four radials centered on the recorder location and for summer conditions for comparisons with visual surveys (Table S5). The probability of detection of P = 0.1 (10th percentile; greatest values, Tables S6 and S7) was used for the detection range visualization and analysis. Detection ranges could not be estimated for SG due to mooring-related noise, therefore the greatest values by species at GS were used for detection range (circle) visualization and analysis at SG.

### Visual surveys

Ship-based visual cetacean sighting surveys are regularly conducted by DFO in BC^19,20^. Observations from three visual surveys around Gwaii Haanas that overlapped with PAM data were used, taking place in 2009–2010, 2018 and 2019 (Fig. 1). The 2009–2010 survey (25 Feb 2009 to 19 Jul 2010) occurred over 10 different legs and the survey design was not systematic (i.e., ships were not following predetermined transects and were closing on whale sightings). The 2018 survey (5 Jul to 4 Sep) and 2019 survey (3–7 Jul) followed a systematic line-transect design detailed in^20^. In 2018 and 2019 surveys, two observers were stationed on the research vessel deck above the navigation bridge, each scanning continuously on either side of the transect line using 7 × 50 Fujinon binoculars. To determine the position of animals sighted, radial distances to sightings were determined using the binocular’s reticles or estimating distance by eye if animals were close to the vessel, and radial angles were measured using electronic angle boards made from digital protractors. An additional observer used Fujinon 25 × 150 MTM pedestal-mounted binoculars to assist the two primary observers with species identifications and group size counts.

### Comparison of acoustic and visual detections

Noise generated by the mooring during periods with tidal current was present during all months and all deployments at SG. This noise was responsible for most fluctuations of the broadband SPL. While most mooring noise occurred below 200 Hz and regularly exceeded the limits of prevailing noise in that frequency band (and rendered both blue whale automated detector and fin whale automated detector ineffective during the first deployment; 2009–2010), it also extended to frequencies up to 8 kHz. At RI, mooring noise occurred primarily from September to January, when storms and high wind periods were more frequent. In contrast, GS recordings were largely free of equipment-related noise. Due to the absence of mooring-related noise, GS was the only site where the contribution of cetacean calls to ambient noise levels was evaluated.

**Figure 2:**
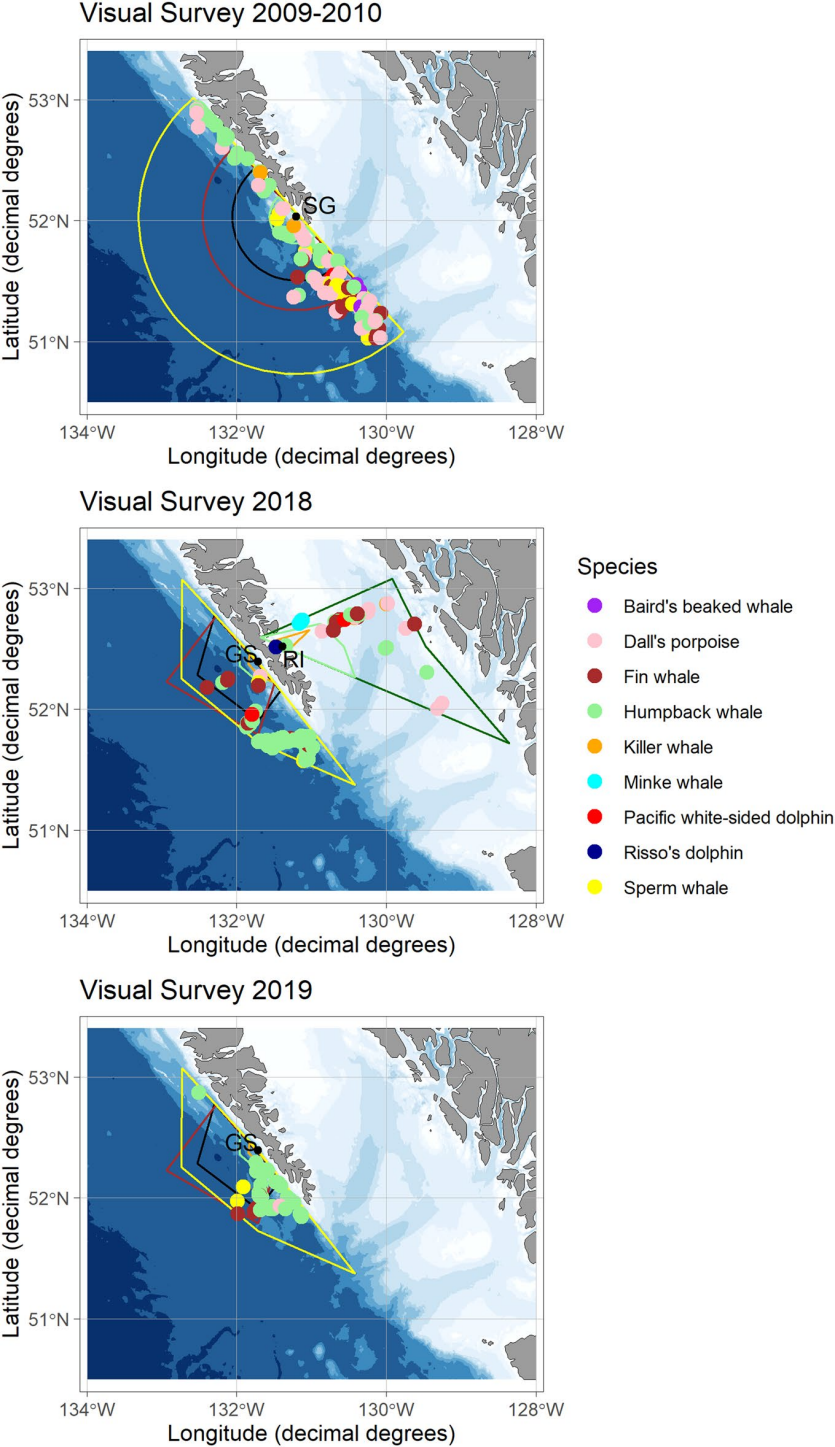
Cetacean sightings by species (colored dots) within the detection range of each hydrophone (black dots) during each summer visual survey, and summer detection ranges (colored lines) for each species in the study area (top: 2009–2010; middle: 2018; and bottom: 2019). Detection ranges of RI for both gray whale and fin whale were identical. Pacific white-sided dolphin had the smallest detection range from RI (< 2.4 km; not visible on the map). Maps were created using the ggOceanMaps package (version 1.26) in R^122^.

To compare cetacean acoustic presence to visual presence at each hydrophone location and corresponding year(s), all visual sightings within the detection range of the species with the largest detection range at each location (sperm whale at GS and SG; gray whale at RI; Fig. 2) were used with the understanding that cetacean species are highly mobile. Time periods that overlapped between acoustic recordings and visual surveys in each of the corresponding detection ranges were used. Because the 2009 recordings started in September, and 2010 recordings overlapped only three days of visual surveys (15–17 July 2010); both July/August 2009 and July 2010 visual surveys were included assuming that the species acoustically detected in 2010 might also have been recorded in 2009 if the recording period had started in July. For each time period, the species detected were catalogued by both methods and those only present in one or the other.

### Environmental parameters (chl-a and SST)

To examine the effects of environmental conditions on whale acoustic detections, correlations between each of SST and chl-a, and cetacean calls by species were examined. Environmental datasets used were 8-day composite SST from the Advanced Very High Resolution Radiometer (AVHRR) at 4-km grid resolution, and 8-day chl-a from the MODerate resolution Imaging Spectrometer on the Aqua platform (MODIS-Aqua) at 4-km grid resolution^21^. Satellite data, focusing on the northeast Pacific Ocean within an area bounded by 130–135° W and 50–55° N, were extracted for the three hydrophone locations. The variable of interest was averaged over 20 × 20 km-square around each hydrophone location to focus on near-mooring cetacean presence, resulting in a single timeline for each variable. Environmental data spanned from 14 Sep 2009 to 17 May 2011 for SG, 11 Jul 2017 to 11 Jul 2019 for GS, and 21 Aug 2018 to 12 Aug 2019 for RI. SST (°C) data were downloaded from the National Oceanic and Atmospheric Administration (NOAA) website^22^. Chl-a (mg/m^3^) data were downloaded from the National Aeronautic and Space Administration (NASA) website (https://oceancolor.gsfc.nasa.gov)^23^. More chl-a data were missing, particularly in winter due to low sun angle. Missing values were replaced with a median value calculated for each site.

For each whale species, 8-day averages for proportion of hours with whale calls, corresponding to 8-day composites for chl-a and SST, were calculated. Times without whale calls were common, therefore the proportion of hours with whale calls were not normally distributed and non-parametric statistical tests were used to quantify the influence of location, chl-a, and SST on proportion of hours with calls for blue whale, fin, humpback, sperm, and Baird’s beaked whales. These whale species were selected based on potential migratory behavior. Baird’s beaked whales were detected only at GS, therefore the influence of location for this species was not tested. A generalized additive mixed model framework (GAMM) was developed using the *mgcv* package^24^ in R Version 4.0.5^25^. Because an 8-day composite for the proportion of hours with whale calls was used, a logarithmic link function with a quasi-binomial distribution was used within the GAMM. Location (SG, GS, and RI) was defined as a random factor, and chl-a and SST were fixed factors. Using package *FSA*^26^ a post-hoc Dunn’s test of multiple comparisons following a Kruskal–Wallis test was conducted on random factors that were statistically significant. All analyses were performed in R Studio (Version 1.4.1106) statistical software platform.

## Results

### Acoustic detection of cetaceans

**Figure 3:**
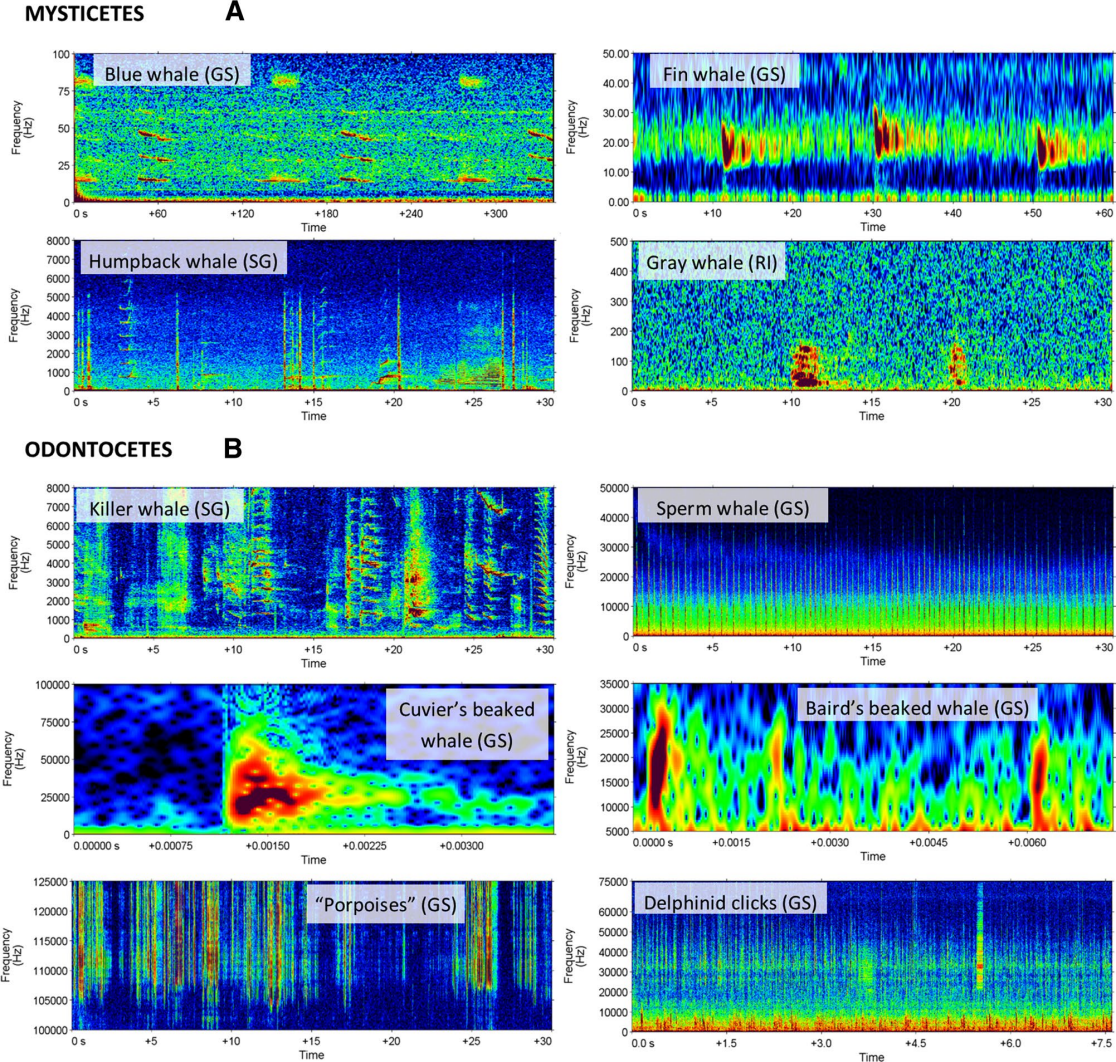
Spectrograms of four mysticetes and six odontocetes calls detected in Gwaii Haanas. For easier viewing, spectrograms (all: Hamming window) are plotted on different time and frequency scales. Blue whale (Gowgaia Slope): infrasonic (**A**–**B**) vocalizations (0.4 Hz frequency resolution, 2 s time window, 0.5 s time step). Fin whale (Gowgaia Slope): 20-Hz pulses (0.25 Hz frequency resolution, 0.3 s time window, 0.03 s time step). Gray whale (Ramsay Island): vocalizations; Humpback whales (SG̱ang Gwaay): song vocalizations; Killer whales (SG̱ang Gwaay): vocalizations (all 2 Hz frequency resolution, 0.128 s time window, 0.032 s time step). Sperm whale (Gowgaia Slope): clicks (64 Hz frequency resolution, 0.01 s time window, 0.005 s time step). Cuvier’s and Baird’s beaked whales (Gowgaia Slope): clicks (512 Hz frequency resolution, 0.26 ms time window, 0.02 ms time step). Porpoises and Dolphin (unidentified delphinid click trains) (Gowgaia Slope): clicks (64 Hz frequency resolution, 0.01 s time window, 0.005 s time step).

Passive acoustic monitoring resulted in the equivalent of 525 continuous-recording days, totalling 4 TB of data (71 day equivalents, 0.19 TB at SG 2009–2010; 96 day-equivalents, 0.25 TB at SG 2010–2011; 166 day-equivalents, 1.56 TB at GS 2017–2018; 163 day-equivalents, 1.53 TB at GS 2018–2019; and 29 day-equivalents, 0.45 TB at RI 2018–2019). Analyses of acoustic files confirmed the presence of four mysticetes species and six odontocetes species or group (delphinid, porpoise) off Gwaii Haanas (see Fig. 3 for selection of calls).

For mysticetes, blue whale vocalizations were manually confirmed in the SG and GS data sets via both systematic and detector validation review of acoustic data. At GS, both A-B vocalizations and audible downsweeps (D-calls) were observed for blue whales. Fin whale vocalizations, including 20-Hz and 40-Hz pulses, were found at all three locations, as were humpback whale vocalizations. Humpback vocalizations consisted of hierarchical songs^27^, and non-song vocalizations including grunts and wops^28^. Gray whale vocalizations were sparsely detected during manual review at RI. No right whale (*Eubalaena japonica*) vocalizations were found during the systematic manual analysis of the recordings at all locations, although undetected right whale tonals could have been missed during bouts with humpback songs. Similarly, no minke whale (*Balaenoptera acutorostrata*) vocalizations were found during the systematic manual analysis of recordings at any location. No sei whale (*Balaenoptera borealis*) vocalizations could be identified with confidence.

For odontocetes, killer whale vocalizations (not identified to the ecotypes) were sparsely detected via automated and manual analysis at all three locations. Sperm whale clicks were common in GS data and detected at SG, but not RI. Cuvier’s beaked whale (*Ziphius cavirostris*) clicks were identified in the GS high-frequency recordings, as were infrequent clicks produced by Baird’s beaked whale (*Berardius bairdii*). High-frequency clicks were identified throughout GS high-frequency recordings and identified as “porpoises”. We are currently unable to confidently differentiate between Dall’s porpoise (*Phocoenoides dalli*), harbour porpoise (*Phocoena phocoena*), and Kogia sp.; therefore, results could include one or more of these species. Recording sampling rates at SG and RI were too low to capture porpoise clicks. Delphinid clicks were identified at RI and throughout the high-frequency recordings at GS. Any whistles detected by the dolphin whistle detector but not detected by the specific killer whale whistle detector was classified as a delphinid whistle. Other than the killer whale, the most common delphinid species in the region are the Pacific white-sided dolphin, northern right whale dolphin (*Lissodelphis borealis*), and Risso’s dolphin (*Grampus griseus*)^13^. Pacific white-sided dolphins produce whistles^29^; however, most of their sounds appear to be burst pulses and clicks^29^. Similarly, Northern right whale dolphins mostly produce clicks and burst pulse sounds^30^. Risso’s dolphins are known to produce clicks, burst pulsed sounds, and whistles^31^. We could not confidently differentiate between dolphin species based solely on these recordings, delphinid whistles at GS and RI could have been mostly produced by Risso’s dolphin, but further analysis is required. Other whistle-producing delphinids are only very rarely encountered in BC and include: common dolphins (*Delphinus delphis* and *Delphinus capensis*), false killer whales (*Pseudorca crassidens*), short-finned pilot whales (*Globicephala macrorhynchus*), striped dolphin (*Stenella coeruleoalba*), and bottlenose dolphins (*Tursiops truncatus*)^13,32^. These species are not likely to have contributed much to the overall delphinid occurrence, but cannot be ruled out without further analysis. Dolphin whistles could not be identified at SG where the recording frequency could not capture these signals.

### Seasonal and diel acoustic vocalization patterns

**Figure 4:**
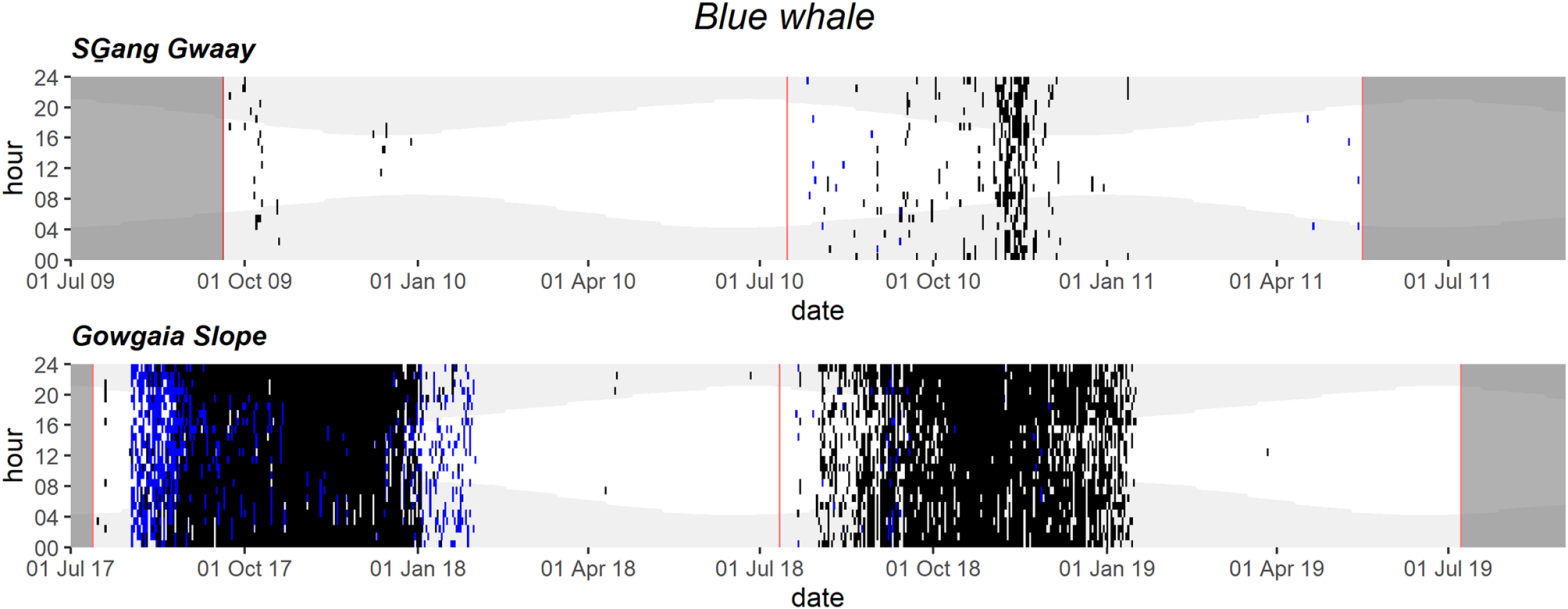
Daily and hourly occurrence of blue whale vocalizations at SG̱ang Gwaay and Gowgaia Slope in 1-h bins. Blue dots indicate automated detections. Black dots indicate confirmed occurrence. Red lines indicate recorder deployment and retrieval dates. Dark grey areas indicate a lack of recordings. Light grey shaded areas indicate hours of darkness.

**Figure 5:**
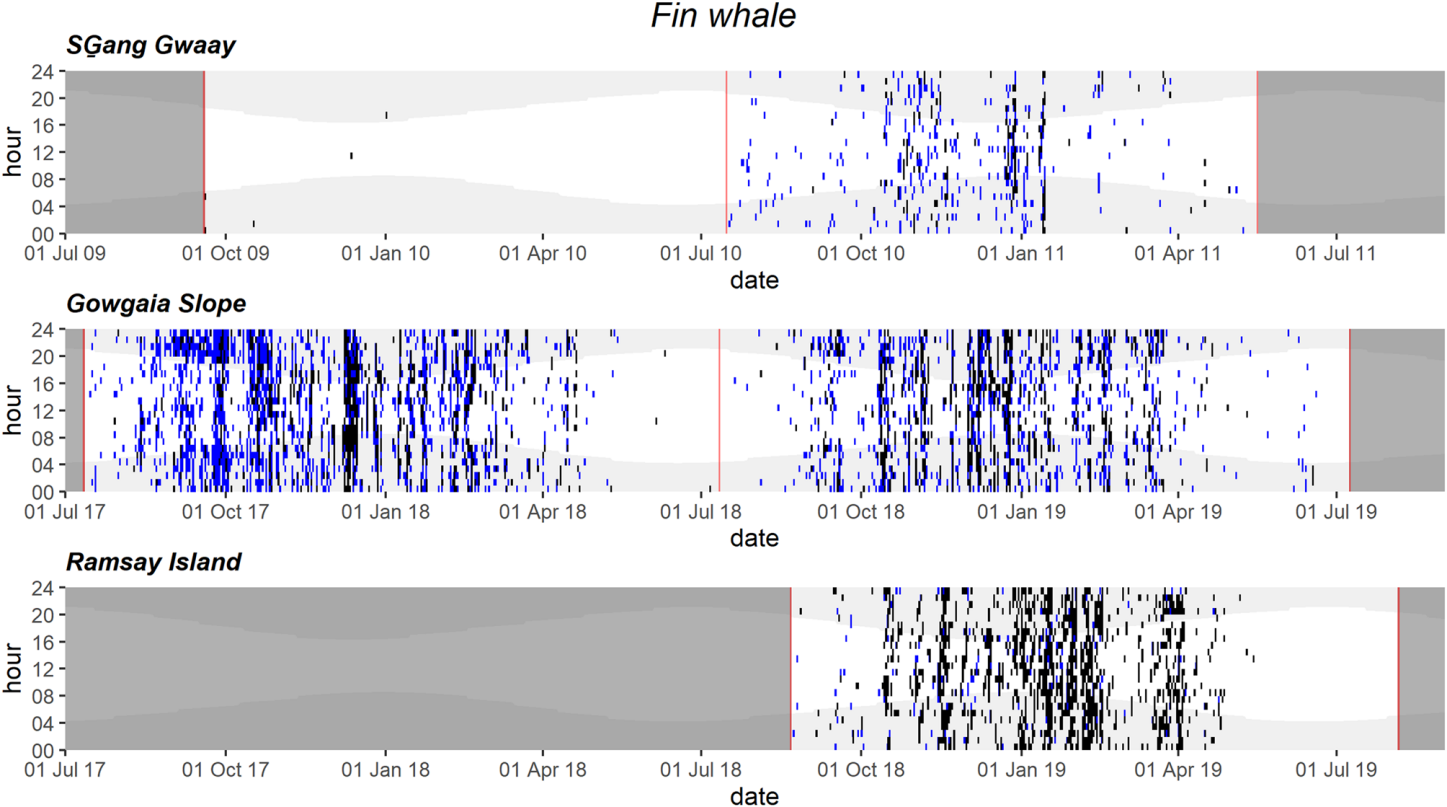
Daily and hourly occurrence of fin whale vocalizations at SG̱ang Gwaay, Gowgaia Slope and Ramsay Island in 1-h bins. Blue dots indicate automated detections. Black dots indicate confirmed occurrence. Red lines indicate recorder deployment and retrieval dates. Dark grey areas indicate a lack of recordings. Light grey shaded areas indicate hours of darkness.

**Figure 6:**
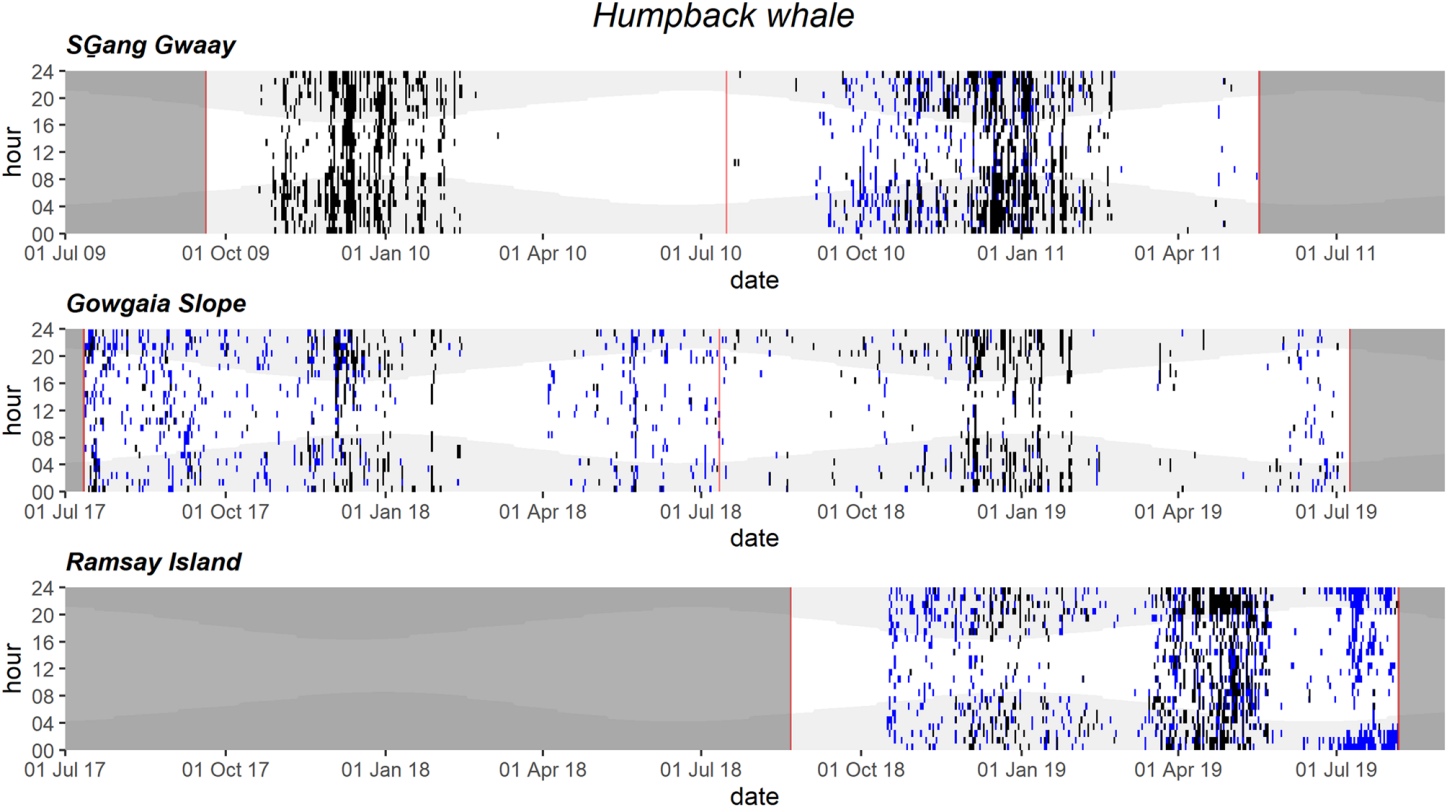
Daily and hourly occurrence of humpback whale vocalizations at SG̱ang Gwaay, Gowgaia Slope and Ramsay Island in 1-h bins. Blue dots indicate automated detections. Black dots indicate confirmed occurrence. Red lines indicate recorder deployment and retrieval dates. Dark grey areas indicate a lack of recordings. Light grey shaded areas indicate hours of darkness.

Blue, fin, humpback, and sperm whales showed strong seasonal acoustic presence (Figs. 4, 5, 6, 8). Humpback whales and delphinids showed strong diel acoustic presence with more activity at night; killer whales showed a similar but more moderate diel pattern (Fig.S4).

Blue whale A–B vocalizations occurred mainly from fall into winter, occurring every day from September to early January in both 2017–2018 and 2018–2019 GS datasets; blue whale calls were also sporadically detected in July and more frequently in August (Fig. 4). Similarly, at SG blue whale vocalizations occurred mainly from October to early January in both 2009–2010 and 2010–2011 datasets. Persistent low-frequency mooring noise in the SG datasets made it challenging for both manual analysts and the automated detector to identify blue whale calls, therefore the SG blue whale occurrence presented here should be taken as an underestimate (Fig. 4).

Fin whale vocalizations were most common from September to April at all three hydrophone locations (Fig. 5). The mooring-related noise at SG also limited our ability to detect fin whale vocalizations manually or automatically, therefore the SG fin whale occurrence presented here should also be taken as an underestimate.

Humpback whale vocalizations occurred in almost all months at GS and RI and peaked in winter when song was present, with RI also having particularly high occurrence of non-song vocalizations during spring 2019 (Fig. 6). Similarly, their calls were most frequent from November to February in both SG datasets, and potential humpback calls occurred sporadically from spring to fall (Fig. 6). Humpback whale vocalizations showed a diel trend during winter months (1 Nov to 28 Feb) with higher acoustic presence at night than during the day (Fig. 6, Fig. S4).

Gray whale vocalizations were sparsely detected at RI on six occasions: one day in January 2019, four days in April 2019, and one day in May 2019. No gray whale calls were detected in the SG or GS datasets.

**Figure 7:**
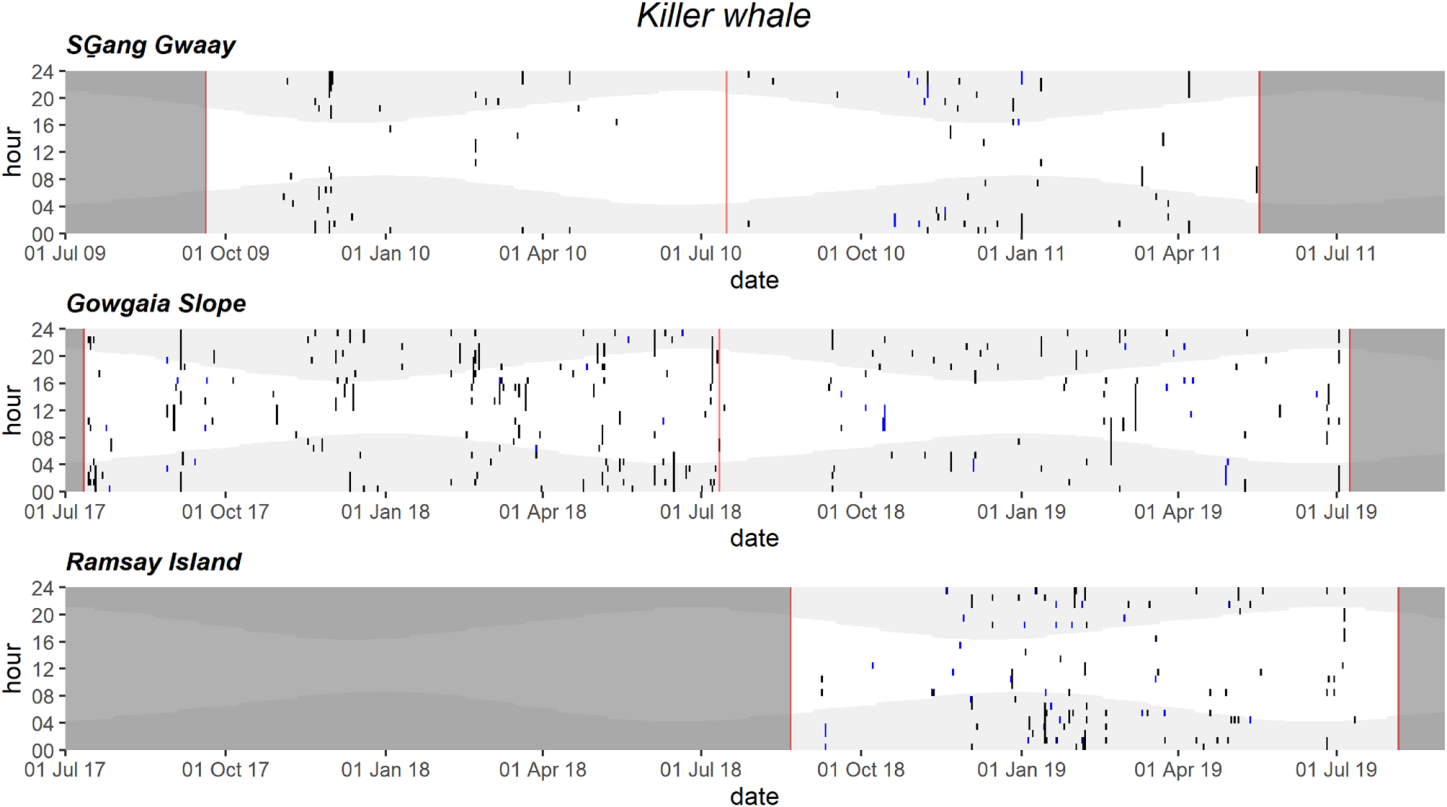
Daily and hourly occurrence of killer whale vocalizations at SG̱ang Gwaay, Gowgaia Slope and Ramsay Island in 1-h bins. Blue dots indicate automated detections. Black dots indicate confirmed occurrence. Red lines indicate recorder deployment and retrieval dates. Dark grey areas indicate a lack of recordings. Light grey shaded areas indicate hours of darkness.

Killer whale vocalizations were relatively sparse and detected in fall and spring throughout all three hydrophone locations (Fig. 7). They were present at GS starting in the fall and into early summer at RI (Fig. 7). Killer whale vocalizations showed a diel trend, with higher acoustic presence at night than during the day (Figs. 7, S4).

**Figure 8:**
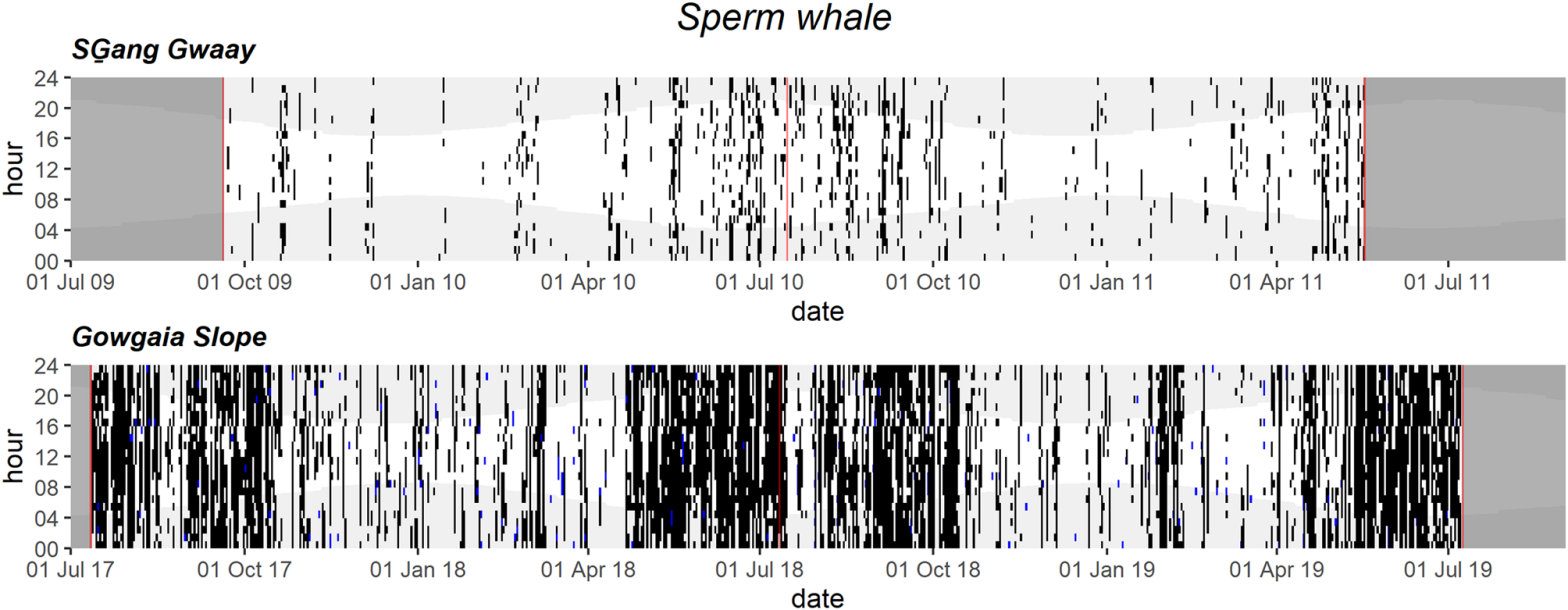
Daily and hourly occurrence of sperm whale vocalizations at SG̱ang Gwaay and Gowgaia Slope in 1-h bins. Blue dots indicate automated detections. Black dots indicate confirmed occurrence. Red lines indicate recorder deployment and retrieval dates. Dark grey areas indicate a lack of recordings. Light grey shaded areas indicate hours of darkness.

Sperm whale clicks were common at both SG and GS throughout the year, with a notable decrease through winter (Fig. 8). Lower recording sampling rate at SG limited manual detection of sperm whale clicks and rendered the automated detector ineffective; therefore, the SG acoustic occurrence is likely underestimated. No sperm whale clicks were detected at RI.

**Figure 9:**
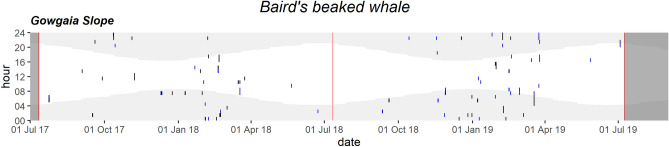
Daily and hourly occurrence of Baird’s beaked whale vocalizations at Gowgaia Slope in 1-h bins. Blue dots indicate automated detections. Black dots indicate confirmed occurrence. Red lines indicate recorder deployment and retrieval dates. Dark grey areas indicate a lack of recordings. Light grey shaded areas indicate hours of darkness.

**Figure 10:**
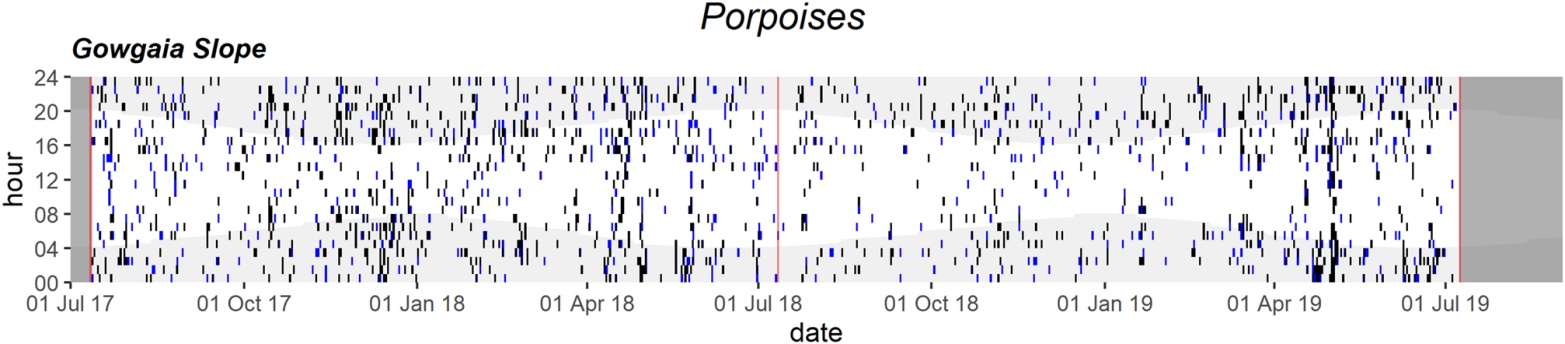
Daily and hourly occurrence of porpoise vocalizations at Gowgaia Slope in 1-h bins. Blue dots indicate automated detections. Black dots indicate confirmed occurrence. Red lines indicate recorder deployment and retrieval dates. Dark grey areas indicate a lack of recordings. Light grey shaded areas indicate hours of darkness.

Cuvier’s beaked whale clicks were manually confirmed mainly in the winter on five occasions in the high-frequency recordings of GS: 24 Nov 2017, 6 Jan 2018, 6 Jun 2018, 3 Nov 2018 and 26 Jan 2019. Baird’s beaked whale clicks were detected throughout the GS recordings mainly during the winter months (Fig. 9). High-frequency clicks attributed to “Porpoises” were also identified through all months of the year (Fig. 10).

**Figure 11:**
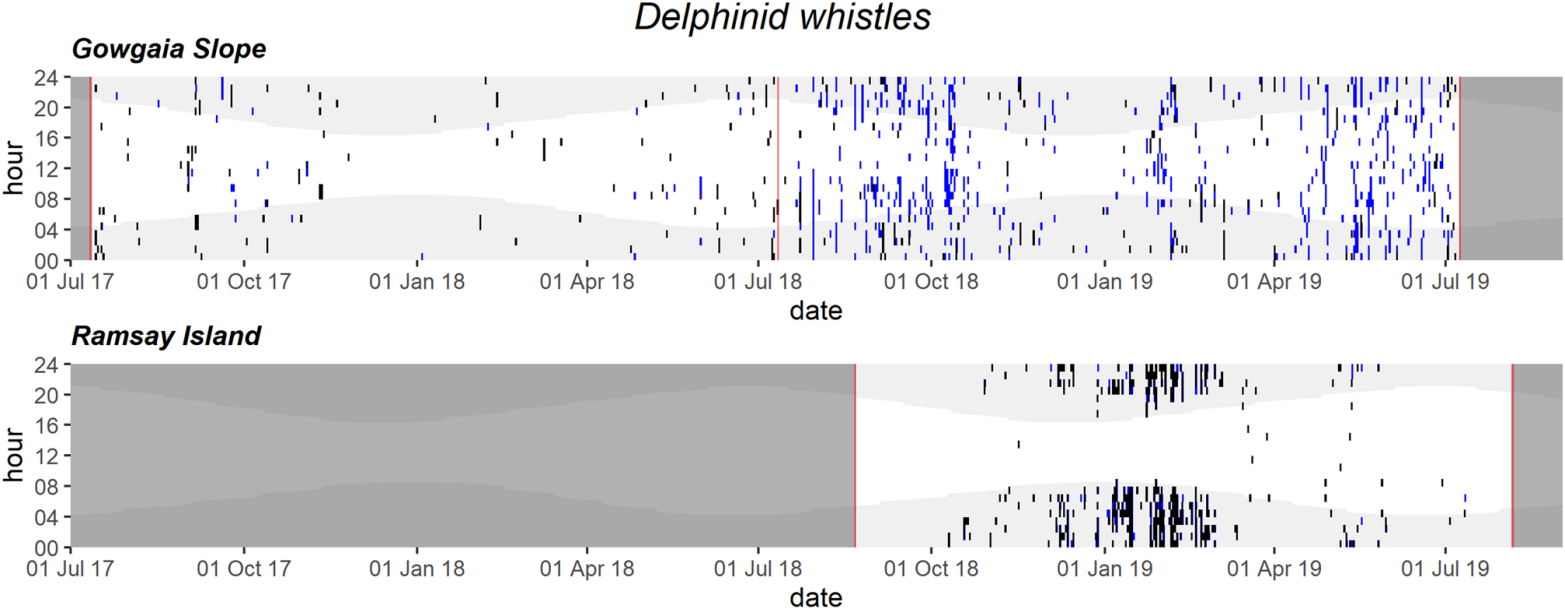
Daily and hourly occurrence of delphinid whistles at Gowgaia Slope and Ramsay Island in 1-h bins. Blue dots indicate automated detections. Black dots indicate confirmed occurrence. Red lines indicate recorder deployment and retrieval dates. Dark grey areas indicate a lack of recordings. Light grey shaded areas indicate hours of darkness.

**Figure 12:**
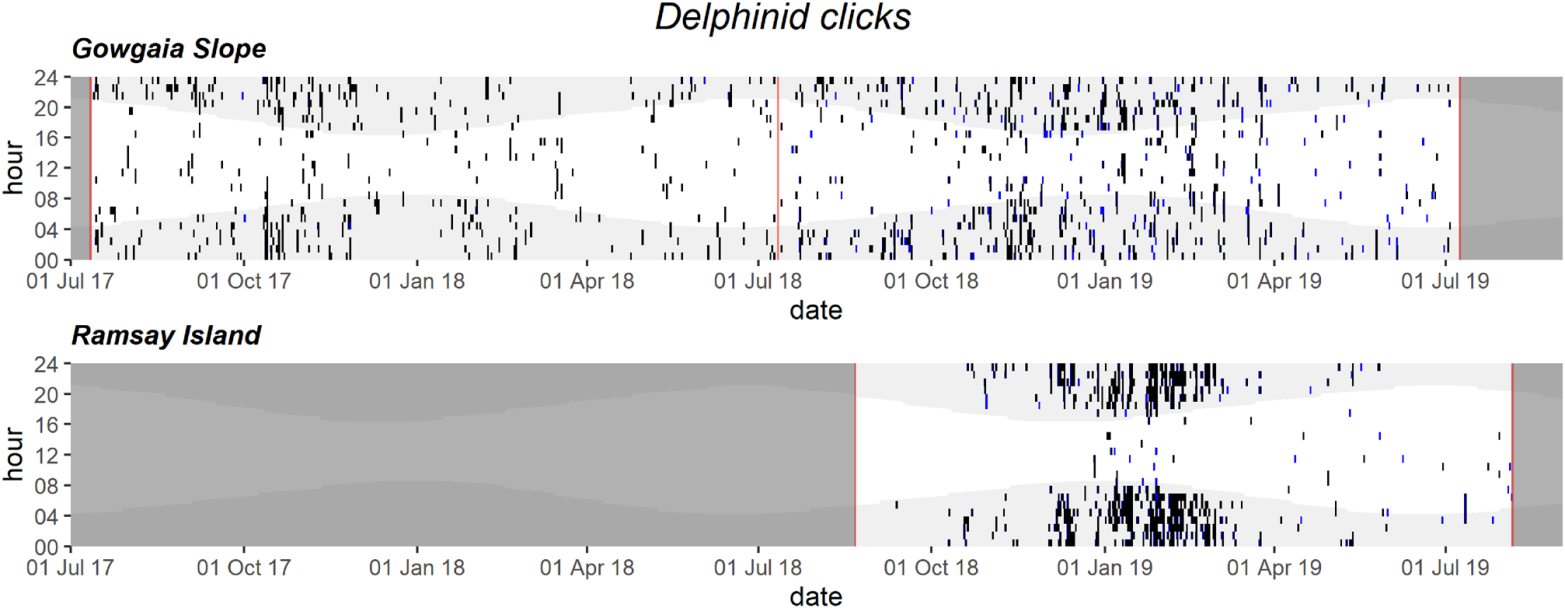
Daily and hourly occurrence of delphinid clicks at Gowgaia Slope and Ramsay Island in 1-h bins. Blue dots indicate automated detections. Black dots indicate confirmed occurrence. Red lines indicate recorder deployment and retrieval dates. Dark grey areas indicate a lack of recordings. Light grey shaded areas indicate hours of darkness.

Delphinid whistles occurred intermittently throughout the year at GS, with less frequency in winter and spring, and occurred mostly in fall and winter at RI (Fig. 11). Delphinid clicks occurred throughout the GS high-frequency recordings, decreasing in spring and summer, and occurred mainly during winter months at RI (Fig. 12). At RI, both delphinid whistles and clicks showed a diel trend, with higher acoustic presence at night than during the day (Figs. 11 and 12, S4).

### Contribution of cetacean calls to ambient noise levels

**Figure 13:**
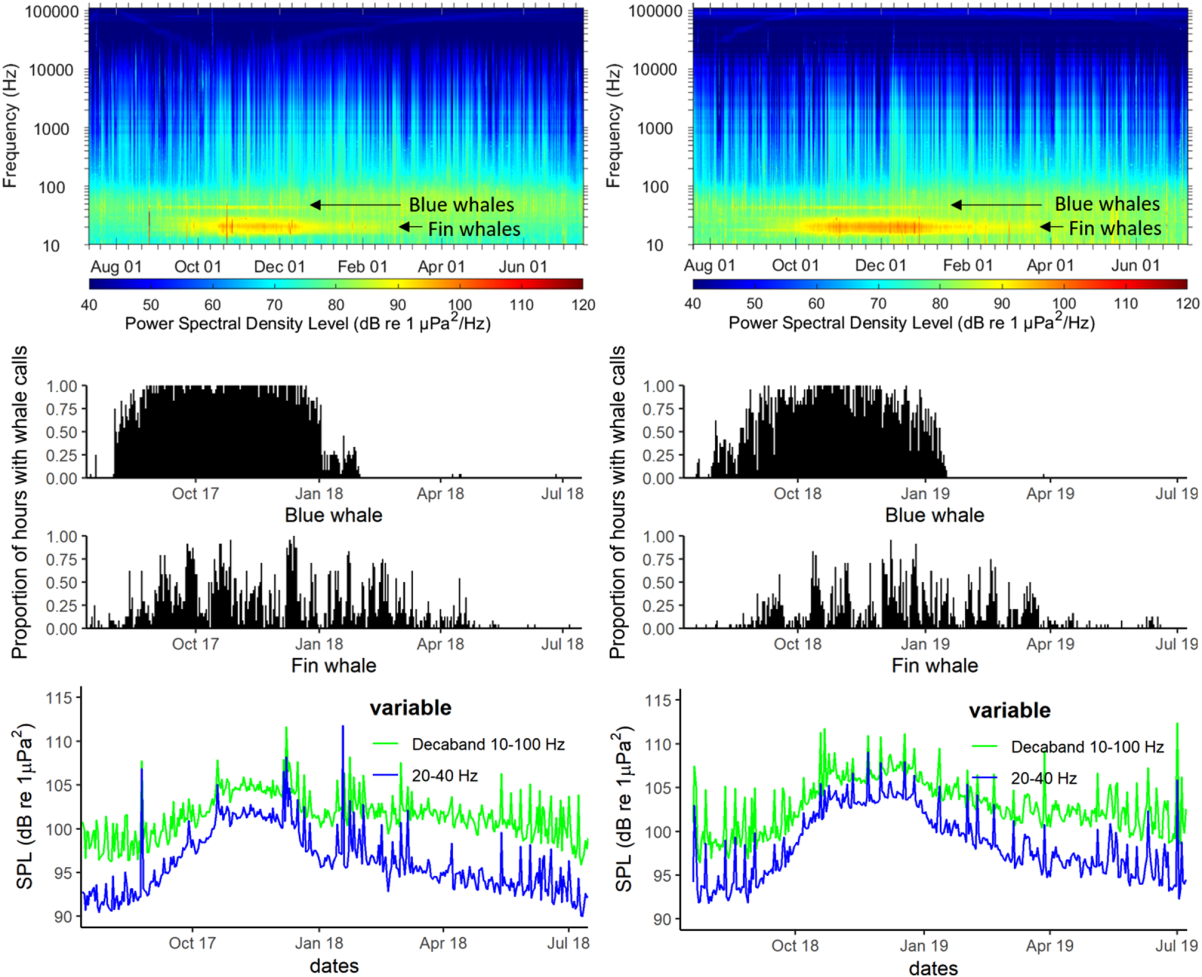
Passive acoustic monitoring data from the Gowgaia Slope hydrophone in 2017–2018 (left) and 2018–2019 (right). Long-term spectrograms showing blue and fin whale call presence (top). Proportion of hours each day with blue or fin whale calls (middle). Sound pressure levels (SPLs) for decaband 10–100 Hz (green) and decaband 20–40 Hz (blue) (bottom).

Two major biological sources contribute to the underwater soundscape at GS in fall and winter. Fin whale presence is evident from the prominent narrow-band noise at 17–27 Hz from October to February/March, corresponding to verified fin whale acoustic presence (Fig. 13). Blue whale presence is evident from the prominent narrow-band noise around 38–55 Hz, which constitutes the louder third harmonic of B calls (generally the most energetic and most commonly recorded), and is found from September to January, correlating with verified blue whale acoustic presence (Fig. 13). Sound pressure levels in decaband 10–100 Hz that represents blue and fin whale calls increased by a maximum of about 5 dB, and in 20–40 Hz that represents mainly fin whale calls increased by a maximum of about 10 dB: these SPLs increased and peaked when both fin whale and blue whale calls were frequently detected in the datasets (Fig. 13).

### Comparison of acoustic and visual cetacean detections

**Figure 14:**
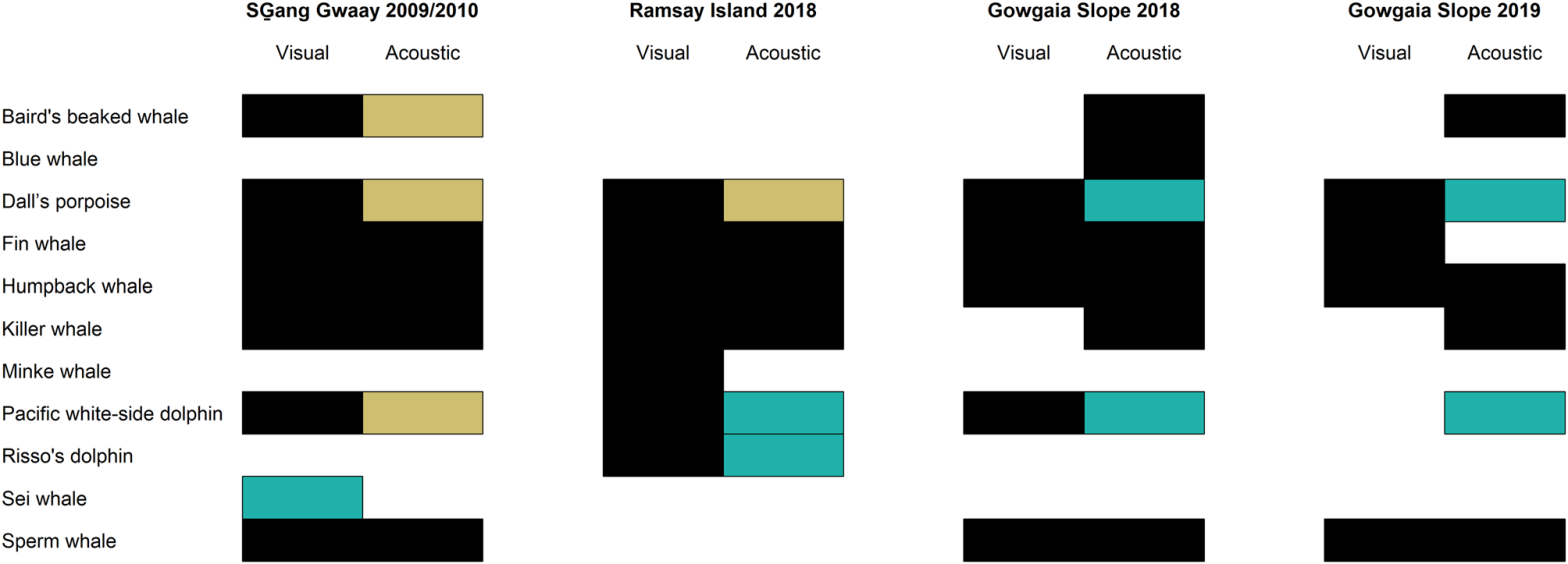
Summary of the visual sightings (black rectangle) from visual surveys (SG̱ang Gwaay: July/August 2009 and July 2010; Ramsay Island: August 2018; Gowgaia Slope July 2018 and July 2019) and cetacean acoustic presence (black rectangle) in the area of hydrophones over the same time periods. Light brown indicates that this species is outside recorder frequency range. Green indicates that the species was likely observed (e.g., sei whale) or cannot be acoustically distinguished to species (porpoise or delphinid groups). *At SG̱ang Gwaay, the 2009 recordings started in September, and 2010 recordings overlapped only three days of visual surveys (15–17 July 2010); thus, 2009 and 2010 visual surveys were included assuming that the species acoustically detected in 2010 might also be recorded in 2009 if the recording period had started in July. At Ramsay Island, the 2018 hydrophone did not get deployed until after the visual surveys were completed.

During the 2009 and 2010 SG visual surveys within the sperm whale detection range of the hydrophone (Fig. 2), 144 cetacean sightings were reported, including seven species identified with confidence, one species likely identified, and five unidentified groups. Over the visual survey period, four cetacean species or groups were acoustically detected (Fig. 14, Table S8). Fin, humpback, sperm and killer whales were detected in both acoustic and visual surveys. The other three confirmed species visually observed—Dall’s porpoise, Baird’s beaked whale and Pacific white-sided dolphin—had high-frequency calls that were outside the range of the hydrophone.

During the August 2018 RI visual survey within the gray whale detection range of the hydrophone (Fig. 2), 42 cetacean sightings were reported, including seven species identified with confidence and three unidentified groups; in the months immediately following the visual survey period when the acoustic recorder was deployed, four cetacean species or groups were acoustically detected (Fig. 14, Table S9). Fin, humpback and killer whales were detected in both acoustic and visual surveys. Dall’s porpoise, Pacific white-sided and Risso’s dolphin sightings were confirmed in visual surveys; delphinid clicks were acoustically present but could not be identified to species, and high frequency porpoise clicks were outside the hydrophone recording range.

During the July 2018 GS visual survey within the sperm whale detection range of the hydrophone (Fig. 2), 83 cetacean sightings were reported, including five species identified with confidence and three unidentified groups; over the visual survey period, eight cetacean species or groups were acoustically detected (Fig. 14, Table S10). Fin, humpback and sperm whales were detected in both acoustic and visual surveys. Dolphin and porpoise clicks were present in the acoustic detections that could not be distinguished to species, while one Dall’s porpoise and one Pacific white-sided dolphin sighting were confirmed in visual surveys. Blue, Baird’s beaked, and killer whales were acoustically detected, but not visually sighted.

During the July 2019 GS visual survey within the sperm whale detection range of the hydrophone (Fig. 2), 54 cetacean sightings were reported, including four species identified with confidence and two unidentified groups; over the visual survey period, eight cetacean species or groups were acoustically detected (Fig. 14, Table S11). Fin, humpback and sperm whales were detected in both acoustic and visual surveys. Dolphin and porpoise clicks were present in the acoustic detections that could not be distinguished to species, while one Dall’s porpoise sighting was confirmed in visual surveys. While Baird’s beaked, and killer whales were acoustically detected along with delphinid clicks not identified to species, no visual sightings of these species were reported.

### Correlation between environmental parameters and acoustic presence

**Figure 15:**
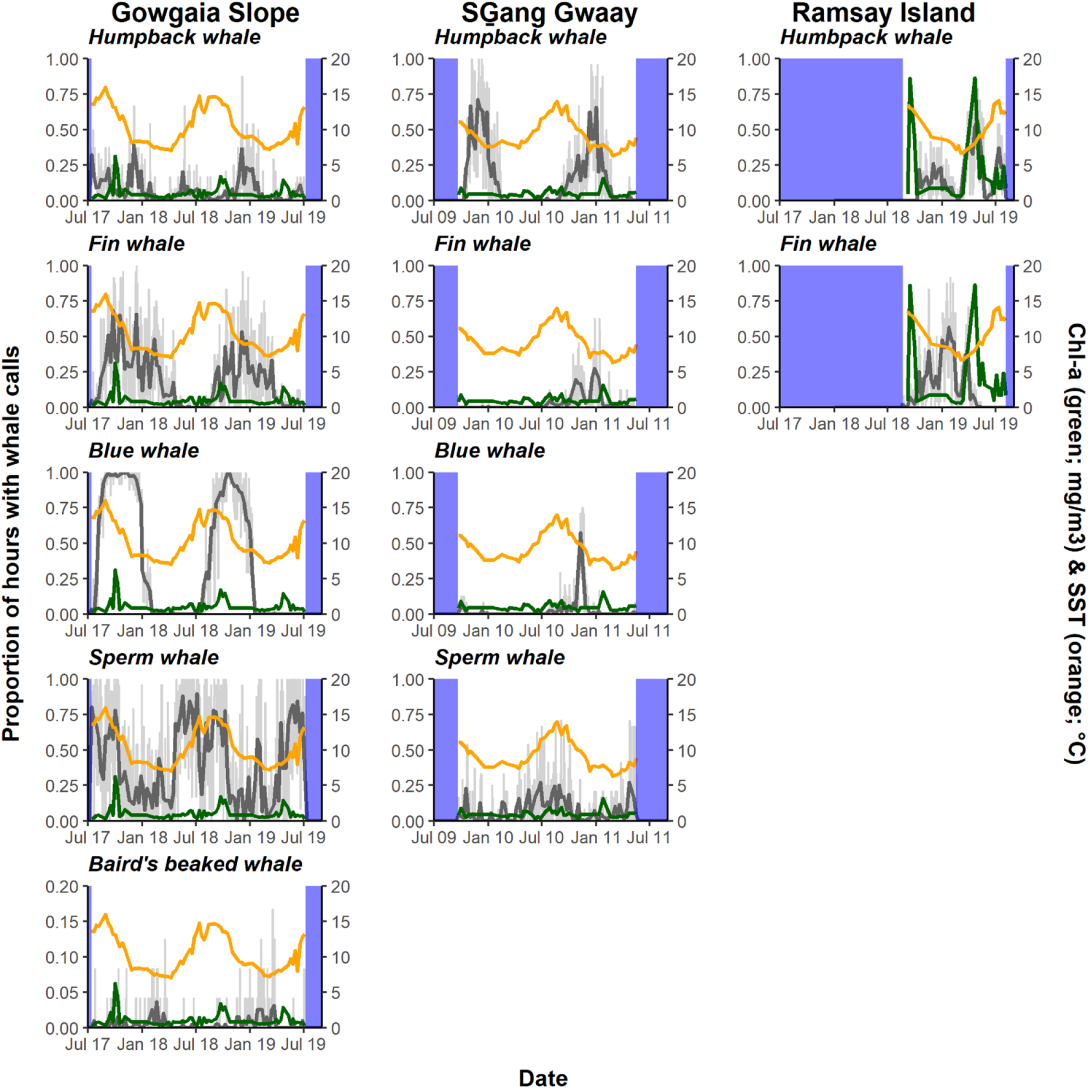
Time series of proportion of hours each day with calls for humpback, fin, blue, sperm, and Baird’s beaked whales (light grey line) and as an 8-day average (composite, dark grey line) over the deployment times for each hydrophone; chlorophyll a (chl-a, green line); and sea surface temperature (SST, orange line) at Gowgaia Slope, SGang Gwaay and/or Ramsay Island. Note that Baird’s beaked whales have a different y-axis scale due to the lower number of detections.

Significant correlations between environmental parameters and/or significant spatial variability in proportion of hours with blue, fin, humpback, sperm, and Baird’s beaked whale calls were found (Fig. 15, Table 2, Figs. S5–S9). Increasing SST correlated with an increasing proportion of hours with sperm whale calls, but a decreasing proportion of Baird’s beaked whale calls. Highest SST values were correlated with the highest proportion of hours with blue whale calls that remained constant until reaching the lowest SST values when proportion of hours with blue whale calls decreased. No significant relationship was found between SST and proportion of hours with humpback or fin whale calls. Increasing chl-a correlated with increasing proportion of blue and humpback whale calls, while no significant relationship was found between chl-a and proportion of hours with fin, sperm and Baird’s beaked whale calls.

**Table 2:** Results of the generalized additive mixed models (GAMMs) of the effect of environmental parameters and location on the proportion of hours with whale calls.

Species	Variable	F	P	% deviance explained	GCV
Blue whale	*Location*	23.08	3.68e–06*	55.1	0.444
	*SST*	4.906	0.000393*		
	*Chl-a*	2.785	0.007263*		
Fin whale	*Location*	14.89	5.42e–07*	21.8	0.187
	SST	0.976	0.325		
	Chl-a	0.252	0.616		
Humpback whale	*Location*	3.847	0.009*	14.1	0.189
	SST	2.221	0.067		
	*Chl-a*	4.469	0.036*		
Sperm whale	*Location*	104.5	< 2.00e–16*	71.2	0.126
	*SST*	9.572	< 2.00e–16*		
	Chl-a	1.868	0.175		
Baird’s beaked whale	*SST*	16.90	0.011*	11.7	0.011
	Chl-a	0.052	0.820		

For each whale species, F-ratios and P-values for each of the three variables (location, SST, and chl-a) are presented, as well as percent deviance explained by the model and generalized cross-validation (GCV) score. Variables with significant P-values (marked with an asterisk) are italicized.

Proportion of hours with whale calls were lowest at GS compared to other locations for humpback whales. Proportion of hours with whale calls were highest at GS compared to other locations for blue, fin and sperm whales. Mooring-related noise at both SG and RI, which was not at GS, could potentially explain some of the location differences. Specifically, the proportion of hours with blue whale calls was higher at GS than SG (Dunn’s test; *Z* = – 2.52, *P* = 0.011); with fin whale calls was higher at GS than at other locations (GS vs. SG: Dunn’s test; *Z* = – 5.35, *P* = 2.64e–7; GS vs. RI: Dunn’s test; *Z* = 4.56, *P* = 1.53e–5); with humpback whale calls was higher at GS than at RI (Dunn’s test; *Z* = 2.47, *P* = 0.041); and with sperm whale calls was higher at GS than at SG (Dunn’s test; *Z* = – 6.65, *P* = 2.90e–11).

## Discussion

Spatial and temporal patterns of cetacean habitat use vary considerably by species and remain relatively understudied in northern BC, including in Gwaii Haanas waters. This study represents the most comprehensive acoustic and visual study of all identifiable cetacean species on the east and west coasts of Gwaii Haanas to date. Passive acoustic monitoring provided unique, year-round, long-term information about cetacean use, seasonality, diel patterns, and geographic distribution that complemented vessel-based visual surveys for the east and west coasts of Gwaii Haanas, a remote protected area on HG in BC, Canada. Vessel access to exposed open ocean areas like the west coast of HG is challenging, particularly in the winter, and the ability to monitor cetaceans by visual surveys off the continental shelf-slope and offshore over multiple years is costly and capacity prohibitive. Over the study period, results indicated acoustic and/or visual presence of twelve cetacean species or species groups, with acoustic seasonal patterns identified for five cetaceans, and acoustic diel patterns for two species and one species group. Implementation and analyses of both survey types over 4 years was made possible by Gwaii Haanas management partners working together. Collaborating to complement summer visual surveys with year-round PAM yielded a more fulsome understanding of cetacean use patterns in and adjacent to Gwaii Haanas that can be used as a ‘baseline’ to monitor future changes, and as a springboard for future research.

### Complementarity of acoustic and visual detection methods

Results from acoustic recordings and visual surveys largely complemented each other in documenting cetacean presence and use on the east and west coasts of Gwaii Haanas. Numerous cetacean species were detected by both acoustic monitoring and visual surveys (i.e., fin, humpback, sperm, and killer whales), while other species were detected by only one of these methods. Visual surveys resulted in many ‘likely’ and ‘unidentified’ sightings (results not shown here), just as acoustic monitoring resulted in some calls by species groups that could not currently be identified to species (e.g., porpoises and delphinid). Acoustic detections confirmed the presence and acoustic use patterns of additional species outside visual survey timeframes: blue and Cuvier’s beaked whales were detected at GS; blue whales at SG, and grey whales at RI. Conversely, some visually-sighted species (minke whales at RI, fin whales at GS in 2019, and potentially a sei whale at SG) were not detected in overlapping PAM datasets.

Absence of visually-sighted species in acoustic recordings could be due to many reasons. Cetaceans may have been in the area but not producing sounds. For example, minke whales were sighted but not acoustically detected here, potentially due to their low calling rate in the study region^33^, and little is known about their seasonal movements in BC^34^. Cetaceans may not have been heard because the PAM area is not within their usual habitat. For example, sei whales are normally found in areas far offshore^35^ and thus may not be detected by coastal hydrophones. Similarity among balaenopterid whale vocalizations outside of the breeding season (notably between non-song vocalizations of blue, fin, and sei whale downsweeps) may also explain the absence of sei whale vocalizations identified with confidence^36^. Hydrophone limitations may result in discrepancies between visual and acoustic surveys. At SG, the 16 kHz recording frequency could not capture delphinid species, beaked whales, porpoises and/or *Kogia* calls; at RI, the 96 kHz recording frequency could not capture porpoises and/or *Kogia*.

Like all survey methods, PAM and visual surveys have strengths and weaknesses that can be complementary and require consideration in data interpretation. PAM is limited by its ability to detect only cetacean calls louder than ambient ocean noise levels, including any mooring related noise, and to acoustically distinguish species-specific calls. Nevertheless, PAM has several major benefits. Moored autonomous acoustic recorders allow PAM at night, during inclement weather and through all seasons^37^ allowing data collection on the seasonal and diel occurrence of many species, including difficult-to-study, cryptic aquatic species, such as porpoises or beaked whales. Since marine mammals spend most of their time below the sea surface, PAM is an important tool that facilitates the study of these animals when they are otherwise visually inaccessible. Visual surveys complemented PAM by identifying species that are sometimes not acoustically detectable or distinguishable, and covering much larger survey areas than within the acoustic detection range of a single recorder for most cetacean species. Nevertheless, visual surveys only observed cetaceans present at the surface within the visual horizon of the survey crew, and confirmed identification was only possible with sufficient proximity to the animal when at the surface. The exact location of animals is known in visual surveys, whereas species-specific detection ranges for each hydrophone deployment means that species with smaller detection ranges (i.e., < 20 km) such as killer whales and dolphins are more likely to be within the boundaries of Gwaii Haanas, whereas those with large detection ranges such as blue, fin and sperm whales could be inside or outside of Gwaii Haanas. Regardless of their physical presence, all of these species contributed to the Gwaii Haanas underwater soundscape.

### Vocalization patterns and environmental correlates by species and group

#### Seasonal patterns of blue whale calls correlate with productivity off west coast

Northeast Pacific blue whales seasonally migrate over large distances, ranging from the waters off Central America to the Gulf of Alaska. This cyclic annual migration is associated with open-ocean feeding at mid- to high-latitudes throughout the highly productive summer and fall, followed by a southbound migration to tropical regions to give birth and mate in the winter and spring. They produce A–B vocalizations throughout the year, at their tropical breeding grounds (song—repetitive bouts of A–B pairs), during migration, and on their feeding grounds^38^, consistent with the daily blue whale A-B vocalizations detected from September to January at GS and sporadic detections at SG on the west coast of Gwaii Haanas. This timing matches that from PAM off the west coast of Vancouver Island within the migration route of the eastern North Pacific population of blue whales^39,40^. Sightings of blue whales off southwestern Gwaii Haanas have been reported within 10 nm (18.5 km) of shore in August 2003, 2005 and 2006 surveys^41^. The absence of blue whales in the summer visual surveys between July 2009 and 2019, and sporadic detection of blue whale calls over the same periods, likely reflects the seasonal movement of blue whales.

Blue whale calls showed a strong seasonality that correlated with environmental patterns, likely driven by prey availability. The most northern ‘chlorophyll concentration regime’, from southeast Alaska to northern BC coastal waters, is characterized by a spring bloom beginning around mid-April that peaks in chl-a between April and June; fall phytoplankton blooms, if they occur, generally take place in September^42^. The increase in blue whale detections off Gwaii Haanas in September coincides with high primary productivity in the fall, likely fueling high zooplankton abundance. In BC, hotspots of the zooplankton *Euphausia pacifica* and *Thysanoessa spinifera*, the most abundant euphausiid species in the northeast Pacific Ocean and preferential prey for blue whales, also peak in September and were highest over the continental slope, particularly off the west coast of HG (data were from April to September^43^). Our finding that blue whale A-B vocalizations are associated with location, SST and chl-a concentration was consistent with other studies that linked blue whale distribution patterns to seasonally productive waters associated with high chl-a^44^. Blue whale calls were also more abundant in regions of high surface chl-a off California and the west coast of Vancouver Island^40^. The proportion of hours with blue whale calls found here matched seasonal SST trends, consistent with correlations found in the North Pacific^38^. Here, blue whale call proportions stayed consistently high from mid-August through December, when the decline in blue whale call proportion was correlated with the lowest SST values. From 2009–2011 and 2017–2019, blue whale calling behavior on the west coast of Gwaii Haanas showed geographic and seasonal patterns with consistent interannual variability.

#### Fin whales call throughout winter on east and west coasts

Fin whale 20-Hz and 40-Hz calls were recorded on the west and east coasts of Gwaii Haanas mainly from September through April, providing further evidence that fin whales are consistently present in BC waters throughout the winter. Fin whale 20-Hz pulses are primarily related to the reproductive period that peaks in December and January in the North Pacific^45–48^, and thus tend to be heard in winter^49^, likely explaining the higher call activity detected from October to March in this study. This October to March peak in calling corresponds well with the peak timing at other acoustic monitoring locations throughout BC^50,51^. Fin whales were also acoustically detected on the east coast at RI, consistent with previous reports and acoustic studies that suggest Hecate Strait’s importance for the species in BC^20,51–55^. Although fewer fin whale calls were detected in spring and early summer (May–July) in Gwaii Haanas, this does not necessarily indicate an absence of fin whales; fin whales are present during spring months around HG^54^. This is also consistent with other studies showing that fin whales reduce or cease making 20-Hz pulses in the summer months^56,57^. Fin whales produce 40-Hz calls primarily during the summer in known feeding areas^57^; those calls were sporadically detected at GS in summer months, and may have been present but not detected at SG and RI due to mooring-related or tidal current-induced flow noise.

Although Stafford et al.^38^ found SST to be the best predictor of fin whale call detections in the North Pacific Ocean, no statistical relationship was found in this study between fin whale acoustic activity and environmental parameters of SST or chl-a in this study. The lack of strong relationship with chl-a may be due to their diet^38^, which consists mostly of euphausiids, but also copepods and small schooling fishes (e.g., herring, Pacific saury^58^). Therefore, fin whales may be less dependent on zooplankton, in contrast to blue whales who feed almost exclusively on euphausiids. Lack of a relationship to environmental parameters could also be explained by the delay between primary productivity and abundance of higher trophic level prey on which fin whales feed. One to four intermediate trophic levels may occur between primary production assessed via remote-sensing and marine top predators like cetaceans^59^. Finally, it is possible that examining predictors for 40 Hz and 20 Hz fin whale calls separately may yield additional insights, because the two call types appear to be used during different behavioural contexts, with 40-Hz calls being associated with feeding behaviour^60^.

#### Humpback whale calls peak in winter on west coast and spring on east coast

Like most other mysticetes, humpback whales exhibit seasonal migrations from high-latitude feeding areas in summer to low-latitude breeding and calving areas in winter. They commonly feed in BC waters from spring to fall and are widely distributed along the coast^19,20,61,62^. Two large concentrations of humpback whales in BC have been observed around HG in southwestern Hecate Strait^52^ and southern Dixon Entrance^63^. The west coast of HG has also been suggested as a relatively high importance humpback whale feeding habitat^63^.

Here, humpback whale vocalizations occurred at both west and east coast Gwaii Haanas hydrophones. The peak in acoustic activity occurred with songs in winter on the west coast at SG and GS when males were singing^27,64^ Humpback whales have been observed singing outside of known breeding grounds in the North Pacific^65–67^ and elsewhere in the world, where they are potentially practicing songs in preparation for the mating season^68^ or potentially mating^69^. This acoustic evidence suggests that humpback whales are regularly present on the west coast until mid-February, although they are also sighted and heard throughout the spring and summer. Vocalizations were detected almost every month of the year throughout the 2017–2019 GS deployments, but infrequently at SG where significant mooring-related noise may also have masked humpback whale calls. During winter months, humpback whale vocalizations showed a diel trend, with more vocalizations produced at night than during the day (Fig. 6, Fig. S4). Similar behaviour was observed in winter near the Gully Marine Protected Area in offshore eastern Canada^70^.

Along the east coast of Gwaii Haanas, humpback whales are commonly sighted^52,53,71,72^, with higher sighting rates in spring and early summer reported in Laskeek Bay, immediately north of Gwaii Haanas^73^. Although calling activity was present in the winter at RI, peak acoustic activities occurred in the spring, coinciding with timing of Pacific herring spawning activities. At RI, increasing humpback whale acoustic occurrence with increasing chl-a concentrations was consistent with other studies showing similar observations for southwestern Hecate Strait^63^, where the highest estimated chl-a concentrations have occurred in Gwaii Haanas^74^.

#### Occasional gray whale calls correspond to annual migrations

Gray whales undertake annual migrations north in spring to summer feeding grounds in the Bering and Chukchi Seas, and south in fall to winter in the breeding and calving lagoons along western Baja California, Mexico^75^. Gray whale vocalizations were detected on a few occasions at RI: once during the winter and mainly during the spring, consistent with previous visual observations of gray whales occurring near RI mainly in April^76^. Their spring presence in Gwaii Haanas corresponds with the Pacific herring spawning season when gray whales spend time in shallow coastal waters feeding on herring spawn (L. Lee, pers. comm.). Lack of detections on the west coast is consistent with results from satellite-tagged gray whales and shore-based surveys, which together provide clear evidence that most gray whales travel through along Hecate Strait and Dixon Entrance as their migratory corridor between Vancouver Island and southeastern Alaska^77^. The sparse occurrences of gray whales in Gwaii Haanas is likely explained by their preference for deeper waters of eastern Hecate Strait rather than shallower waters on the western side^77^. During the northward migration on the west coast of Vancouver Island at Flores Island gray whale calls are detected from February to early May with a peak in mid-March^78^. The January detection at RI would correspond to the southward migration, which indicates that at least some whales use the same corridor for the southbound migration. This timing is consistent with the greatest prevalence of calls heard in early January at Flores Island^78^.

#### Higher spring–summer and lower winter sperm whale calls suggest migration timing

Sperm whale clicks were common on the west coast throughout the year, with highest activity in spring and summer, and a notable decrease through winter. Their year-round acoustic presence was not surprising, given that mature sperm whales and small groups of younger males are known to forage year-round at higher, more productive latitudes, to build up size and social maturity^79–82^. Mature males then migrate to low latitude breeding grounds in warm waters on an unknown schedule^83^. In contrast, females and immature whales tend to stay in tropical to temperate waters throughout the year^83^. Mixed sex social groups have been identified as far north as 50°N in the North Pacific during the summer^84^.

The seasonal pattern off Gwaii Haanas could represent movements of sperm whales into the area during the spring and summer and out of the area during the winter, an increase in foraging during the warmer seasons, or some breeding activity in the spring. Given that males are spatially segregated by size, with larger males at higher latitudes^79^, it might suggest that some males are moving between the Gulf of Alaska/BC and southern areas. Another hypothesis is that females travel to this area to increase their encounter rates with stronger more mature males^35^. Reproductive data from sperm whales taken off BC suggests they were mating in these northern waters in late spring (April–May), before females moved farther offshore to calve in summer (July–August)^61^. Both males and females produce clicks, therefore either or both sexes may be present off Gwaii Haanas. Sperm whale clicks were absent on the east coast, consistent with their known ecology and visual sightings reported only along the west coast of HG^19^. Sperm whale acoustic occurrence was lower in winter during cooler SST, consistent with the positive relationship between sperm whale occurrence and increased ocean temperatures in the Gulf of Alaska^85^.

#### Intermittent presence of killer whales

Killer whale vocalizations occurred intermittently throughout the year, with weeks of higher vocalization activity occurring in fall-early winter and spring on the west coast, and in winter, spring and summer on the east coast. These patterns are consistent with visual observations throughout the east and west coasts of Gwaii Haanas^76^. Although a detailed analysis of killer whale call types was not conducted for this study, the vocalizations included all ecotypes: resident (fish-eating), west coast transient (Bigg’s; mammal-eating), and offshore (largely shark-eating) killer whales^86^, which have all been documented in this region previously^87–90^, but visual observations of Bigg’s killer whales are the most commonly reported in HG waters (L. Lee, pers. comm.).

A diel trend was observed in the vocalizations, with higher nighttime acoustic presence than during the day. This trend is similar to patterns for Bigg’s killer whales in the Bering Sea^91^, which could suggest that at least some of our recordings are Bigg’s ecotype. Bigg’s killer whales, which feed on other marine mammals^92^, vocalize much less frequently than northern residents^93–95^, presumably to avoid detection by their prey. They travel in small groups over a wide geographic range (California to Alaska) including inshore and offshore waters of HG^19^, and are not confined to any single area^96,97^. An ongoing study with detailed analysis of killer whale call types should provide further information (Ford et al. in preparation).

#### Acoustic presence of rarely-sighted Cuvier’s and Baird’s beaked whales

The North Pacific is inhabited by at least ten species of beaked whales. Unfortunately, information on the abundance and distribution of all these species is scarce and limited due to their highly elusive behavior, with short surface intervals and prolonged deep dives^98^, and a small number of visual sightings and strandings. Nevertheless, they produce echolocation signals which appear to be species-specific^99^. Baird’s and Cuvier’s beaked whale clicks confirmed their presence on a few occasions in the west coast GS high-frequency recordings, mainly during winter months. Proportion of hours with Baird’s calls decreased with increasing SST, also suggesting more frequent occurrences in winter. Their presence in BC has also been reported in spring–summer visual surveys^100^, and BC historical whaling catches with peak numbers caught in August^101^. Although Cuvier’s beaked whales have been reported in BC mostly around HG and the west coast of Vancouver Island^19,101,102^ the absence of detections at RI, might be possibly due to recorder location, the lower sampling rate and/or mooring-related noise. Clicks from *Mesoplodon* spp. were not identified even though two species, *Mesoplodon stejnegeri* and *Mesoplodon carlhubbsi*, are known to occur along the BC coast from sightings and strandings (^13,101^; DFO Cetacean Research Program unpublished data).

#### Year-round presence of porpoises on the west coast

High-frequency porpoise clicks at GS include possibly Dall’s and/or harbour porpoises, and/or potentially *Kogia* species, documenting their year-round presence off the HG west coast. Dall’s porpoises are found in cool temperate pelagic North Pacific waters between 32°N and 62°N^103^. In the northeastern Pacific, harbour porpoises inhabit cool temperate coastal waters^104^ from Point Conception, California, to the Bering Sea, preferring shallow (< 100 m depth) nearshore waters^105^, and are also found off the continental shelf. They occur year-round in BC throughout nearshore and shelf waters, moving seasonally inshore to offshore, likely in response to abundance and distribution of prey^52^. Two species of *Kogia* are found in BC and believed to forage for cephalopods in mid- to deep-waters^106^. All four species differ in mean characteristics of their clicks, but have considerable overlap in acoustic bandwidth^107–109^. The difficulty in acoustic species identification for porpoises and *Kogia* due to high variability of presumed *Kogia* clicks has been recently demonstrated^110^, therefore further work is required to distinguish these species acoustically.

#### More dolphin calls at night suggest prey-driven movement patterns

Delphinid whistles and clicks, most likely from Risso’s, Pacific white-sided and/or Northern right whale dolphins, occurred year-round at GS with a decrease in whistles over winter and early spring, while at RI these occurred mostly from winter into early spring with few acoustic detections in summer. Northern right whale dolphins have been sighted in BC including by HG^13^. Pacific white-sided dolphins are year-round residents of both pelagic and nearshore waters in BC, and one of the most frequently sighted during visual surveys in offshore waters, including HG, and in coastal inlets^19^. Risso’s dolphins are a large dolphin distributed world-wide in tropical and temperate waters; in the northeastern Pacific, BC waters appear to be the northernmost extent of their range^19,111^. Occasional but consistent summer sightings of Risso’s dolphins in Gwaii Haanas and HG west coast are reported, sometimes in large groups of over 100 individuals, including near RI; pods of Risso’s with young juvenile pink-faced individuals have also been reported in early spring on the east coast of Gwaii Haanas (L. Lee, pers. comm.).

Dolphin whistles and clicks showed a clear diel pattern on the east coast of Gwaii Haanas, with more activity at night than during the day, consistent with findings for several dolphin species^112,113^, including Risso’s^114^ and Pacific white-sided dolphins^115^. These studies showed night-time foraging on diel vertical-migrating mesopelagic squid and myctophids that are more accessible at night when they migrate to shallower waters. Whether the lack of clicks and whistles during the day reflects absence of dolphins from the hydrophone range or presence of non-vocalizing animals is uncertain. Dolphins can readily move in and out of hydrophone range within a day, as shown by spinner dolphins off Hawaii that followed both vertical (shallow-deeper waters) and horizontal (inshore-offshore and/or alongshore) diel prey behaviour^112^. Thus, dolphins producing clicks and whistles at RI may be following diel vertical and inshore-offshore patterns driven by spatio-temporal prey dynamics^116^.

### Cetacean calls in the ocean soundscape

Worldwide, whales are the primary biological sound source in the oceans, and the third major sound source overall, following ships and wind^117^. Off the Gwaii Haanas west coast at GS, whale call activity peaked in winter, increasing SPLs by approximately 5 dB for decaband 10–100 Hz and 10 dB for band 20–40 Hz (Fig. 13). Blue whales (38–55 Hz), fin whales (17–27 Hz), and humpback whales (50–2000 Hz) to a lesser extent, were the main contributors to the winter soundscape. Blue whales have a great acoustic power^118^, and their call repertoire includes intense, low frequency, long-duration continuous calls^119^. Increased fin whale singing during the peak of breeding season also contributed to higher levels of ambient ocean noise off the west coast in winter. Both species produce high-intensity calls, with the average blue whale call source level of 178 dB re:1 µPa-1 m (A call) and 186 dB re:1 µPa-1 m (B call) over 10–110 Hz^120^, and the average fin whale 20-Hz call source level at 189.9 ± 5.8 dB re:1 µPa-1 m over 13–35 Hz^121^.

## Conclusion

Comparing overlapping periods of visual surveys and acoustic monitoring confirmed presence of 12 cetacean species/species groups within the study region. Seasonal patterns were identified for blue, fin, humpback, grey and sperm whale acoustic signals. Killer whale and delphinid acoustic signals occurred year-round on both the east coast and west coast of Gwaii Haanas and showed strong diel variation. Cuvier’s and Baird’s beaked whale and porpoise clicks, were identified in high-frequency recordings on the west coast. Blue, fin, and humpback whales to a lesser extent, were the main contributors to the winter soundscape on the west coast. The analysis of effects of environmental conditions on whale acoustic detections contributed ecological insights into the presence and acoustic behavior of many cetacean species and groups in these waters. Cetaceans spend most of their time below the ocean surface, highlighting the importance of passive acoustic monitoring, in conjunction with visual surveys, as necessary tools to facilitate understanding and mapping their year-round spatial and temporal distributions. Year-round PAM data complements visual surveys that often occur in summer when weather and sea state are better for cetacean observations; PAM provides insights into seasonal and diel patterns of acoustic use throughout the year, while visual surveys can be used to estimate the density and abundance of animals and confirm spatial distributions. For Gwaii Haanas, collaborations between management partners and external experts were and continue to be critical to successful implementation of the marine monitoring program, including the use of PAM and visual surveys for monitoring cetacean acoustic use and ocean noise.

## Supplementary Information

Supplementary Information.

## Data Availability

The acoustic data used for this study is available per request to Dr. Lynn Lee (Parks Canada, lynn.lee@pc.gc.ca). The visual data used for this study is available per request to Dr. Thomas Doniol-Valcroze (Fisheries and Oceans Canada, Thomas.Doniol-Valcroze@dfo-mpo.gc.ca).
