# Supplementary material for: Acoustic and visual cetacean surveys reveal year-round spatial and temporal distributions for multiple species in northern British Columbia, Canada
Source: Sci Rep. 2022 Nov 10;12:19272. doi: 10.1038/s41598-022-22069-4 (PMC9649617; doi:10.1038/s41598-022-22069-4)
Supplement: Supplementary file 1 — Supplementary Information. [file 41598_2022_22069_MOESM1_ESM.pdf]

Supplemental Material

Acoustic and visual cetacean surveys reveal year-round spatial and temporal distributions for multiple species in northern British Columbia, Canada

Héloïse Frouin-Mouy[1*‡†], Xavier Mouy[1‡°], James Pilkington[2], Elizabeth Küsel[3], Linda Nichol[2], Thomas Doniol-Valcroze[2], Lynn Lee[4]

1 – JASCO Applied Sciences Ltd, Victoria, BC, Canada

2 – Fisheries and Oceans Canada, Cetacean Research Program, Pacific Biological Station, Nanaimo, BC, Canada

3 – JASCO Applied Sciences Inc, Vancouver, WA, USA

4 – Gwaii Haanas National Park Reserve, National Marine Conservation Area Reserve, and Haida Heritage Site, Skidegate, BC, Canada

*: hfrouinmouy@uvic.ca

‡: These authors contributed equally

†: Currently at University Corporation for Atmospheric Research (UCAR|CPAESS), under contract to National Oceanic and Atmospheric Administration, National Marine Fisheries Service, Southeast Fisheries Science Center, Miami, FL, United States

°: Currently at Integrated Statistics, Inc., under contract to National Oceanic and Atmospheric Administration, National Marine Fisheries Service, Northeast Fisheries Science Center, Woods Hole, MA, United States

1 Passive Acoustic Monitoring

1.1 Cetacean call detections and validation

Information regarding cetacean species that frequent the waters of Gwaii Haanas, their status under the Species at Risk Act (SARA) and the Committee on the Status of Endangered Wildlife in Canada (COSEWIC), and their sounds targeted by the manual and automated analysis are provided in Table S1.

Table S1. Cetacean species off Gwaii Haanas waters, their status under the Species at Risk Act (SARA) and the Committee on the Status of Endangered Wildlife in Canada (COSEWIC), and their sounds targeted by the manual and automated analysis.

Species	Designation	Vocalization types	Reference
Blue whale	Endangered (COSEWIC, SARA)	A and B vocalizations	1
Fin whale	Threatened (COSEWIC, SARA)	20 Hz and down-sweeps (40 Hz)	2
Sei whale	Endangered (COSEWIC, SARA)	Audible down-sweeps	3
Minke whale	Not at risk	Boings	4
Humpback whale	Special concern (COSEWIC)<br>Threatened (SARA)	All vocalizations	5,6
Right whale	Endangered (COSEWIC, SARA)	Up calls	7
Gray whale	Special concern (COSEWIC, SARA)	All vocalizations	8
Killer whale	Northern resident (Threatened: COSEWIC, SARA)<br>Transient (Bigg's; Threatened: COSEWIC, SARA)<br>Offshore (Threatened: COSEWIC, SARA)	Whistles and clicks	9,10
Sperm whale	Not at risk	Clicks	11
Short-beaked Common dolphin sp.	Not at risk	Clicks	12
Long-beaked common dolphin	Not at risk	Clicks	12
Risso's dolphin	Not at risk	Clicks	13
Northern right whale dolphin	Not at risk	Clicks	14
Pacific white-sided dolphin	Not at risk	Tonals and clicks	13
Short-finned pilot whales	Not at risk	Clicks and whistles	15-17
False killer whale	Not at risk	Clicks	15
Baird's beaked whale	Not at risk	Clicks	18,19
Stejneger's beaked whale	Not at risk	Clicks	18
Blainville's beaked whale	Not at risk	Clicks	18
Cuvier's beaked whale	Not at risk	Clicks	18,20
Hubb's beaked whale	Not at risk	Clicks	21
Dwarf sperm whale	Data deficient	Clicks	22
Pygmy sperm whale	Not at risk	Clicks	22
Harbour porpoise	Special concern (COSEWIC, SARA)	Clicks	23,24
Dall's porpoise	Not at risk	Clicks	23

Northeast Pacific blue whales produce low-frequency, high-intensity calls, including A and B calls, which are 15–20 s long, often form sequences called songs, and are suspected to be only produced by males [1].

Blue whales also produce D calls that are shorter than the A and B calls (<5 s), have sharp sweep rates between 95 Hz and 45 Hz, and are produced by both males and females [25,26]. Fin whale vocalizations most often consist of long, patterned sequences of 20 Hz notes [27], but also of less frequent downsweeps in the higher frequency range (90 to 40 Hz) [28]. Humpback whales produce famous "songs" [29] as well as other vocalizations and percussive sounds, referred to as "social sounds" [30]. In BC, humpback whale tonal bubble feeding calls have also been documented [31]. Gray whales produce a variety of calls, including pulses and bonging signals, low-frequency moans, grunts, and subsurface exhalations, concentrated below 1500 Hz [32]. While most studies on gray whale sounds have been conducted in calving/breeding lagoons, recent studies showed that gray whales are also highly soniferous off BC while migrating (e.g., [8]). In the Pacific Ocean, minke whales have been recorded making "boing" sounds [4]. Both north Pacific right whales (NPRW) and sei whales produce highly stereotyped and unique calls, up-calls [7] and downsweeps [33], respectively, that are used for acoustic detection. NPRW also produce impulsive gunshot calls that, given their basic structure and non-uniqueness, are harder to use for PAM studies [34].

Killer whales produce three types of sounds: clicks, whistles, and pulsed calls. Clicks are short pulses that usually occur in a series, also called click trains. Click durations range from 0.1 to 25 ms [10]. Whistles are single narrow-band tones in the 1.5 to 18.0 kHz frequency band. They have little harmonic structure and their duration ranges from 50 ms to 12 s [10]. Pulsed calls are harmonically structured with frequency ranges between 80 Hz and 12 kHz [35]. Echolocation signals from beaked whales are mostly frequency-modulated upsweep pulses, which appear to be species-specific and distinguishable by their spectral and temporal features [18]. Cuvier's beaked whales produce clicks with a centroid frequency of ~40 kHz that often had a characteristic C-shaped contour (Figure 3; [20] and [18]). Clicks believed to be produce by Baird's beaked whales were sloped (characteristic of beaked whale clicks, Figure 3) with a centroid frequency of 20–28 kHz and an inter-click interval of 0.2–0.3 s [18,19]. Dall's and harbor porpoises, as well as dwarf sperm whales, produce clicks in the peak frequency range between 119–143 kHz [22,23], making it more difficult distinguish their calls by species. Pacific white-sided dolphin clicks have amplitude maxima at frequencies of 22.2, 26.6, 33.7 and 37.3 kHz, with distinctive low amplitude notches or valleys at 19.0, 24.5 and 29.7 kHz [13].

Automated analysis consisted of running three different types of detectors to identify cetacean calls based on known acoustic characteristics of calls by species or group of species. Blue, fin, and minke whales were detected using a spectrogram correlation approach based on [36] using time-frequency masks corresponding to the calls found in the North Pacific. The detector was configured to detect blue whale A, B and D calls, fin whale 20Hz pulses and 40 Hz downsweeps, and minke whale boing sounds. Humpback and killer whales were detected using the random forest-based classification approach described in [37] and [38]. Whistles and clicks from other odontocetes were detected using the general purpose contour detector described in [39].

Manual analysis was performed in two ways. The first approach consisted of performing a 2.5% systematic manual analysis for all deployments and each channel (Supplementary Table 2). The systematic review resulted in 36 min per day (2.5% of mins in a day) being reviewed for each data set. The analysts visually and aurally examined each selected recording snippet using spectrograms created in the PAMlab software (JASCO Applied Sciences). Annotations were created to indicate marine mammal presence in each snippet. A lack of annotations was indicative of species absence within the detection range or species not vocalizing. The second manual analysis approach, described in [40], was a manual review focused on the 1% of acoustic data files that had automatic marine mammal detections. All files selected for manual validation were reviewed aurally and visually using spectrograms in PAMlab. This second approach was specifically used to optimize the parameters of the automatic detectors (i.e., confidence thresholds and minimum number of detections accepted per file), and to calculate their performance for each location and time period. Although the detectors classify each specific signal, we only validated the presence or absence of species at the file level (one file corresponding to acoustic data recorded from one channel and one duty cycle, see Table 1). Presence of a species was defined by the presence of one or more detections for that species in a file. Acoustic signals were only assigned to a species if an analyst was confident enough in their assessment. In addition to the 1% of files selected for validation, every automated detection of minke,

killer, and right whale was manually reviewed. All automated detections of blue and fin whale were also manually verified between 1 Mar and 1 Aug to confirm their daily presence.

Table S2. Sampling protocol used for the systematic analysis.

Location	Channel (kHz)	Recording snippet duration (s)	File selected for analysis
Gowgaia Slope (2017 & 2018)	16 kHz channel	90	Every fourth
	250 kHz channel	45	Every second
Ramsay Island (2018)	96 kHz channel	90	Every file
SG̱ang Gwaay (2009 & 2010)	16,384 Hz channel	90	Every second

Performance of the detectors was quantified using the precision (P) and recall (R) metrics defined by:

$P = \frac{TP}{TP+FP}$ (S1), $R = \frac{TP}{TP+FN}$ (S2)

where TP is the number of true positives, FP the number of false positives, and FN the number of false negatives, calculated based on the presence/absence of species for each file reviewed with the second manual analysis approach. We calculated P and R for each species and each deployment.

Where the number of validated files was too low, and/or the overlap between manual and automated detections was too limited to calculate P and R, and/or when P < 0.75, automated detections were ignored and only validated results were used to describe the acoustic occurrence of a species (Table S3).

Table S3. Final performance of the automated detector for each species, location and year

Species	Location	Year (deployment)	Precision	Recall
Blue whale	SG̱ang Gwaay	2009	NA	NA
		2010	1	0.35
	Gowgaia Slope	2017	0.93	0.62
		2018	1	0.49
Fin whale	SG̱ang Gwaay	2009	NA	NA
		2010	0.85	0.68
	Gowgaia Slope	2017	0.90	0.90
		2018	0.86	0.75
	Ramsay Island	2018	1	0.84
Humpback whale	SG̱ang Gwaay	2009	0.95	0.83
		2010	0.97	0.85
	Gowgaia Slope	2017	0.79	0.81
		2018	0.91	0.76
	Ramsay Island	2018	0.75	0.75
Killer whale	SG̱ang Gwaay	2009	NA	NA
		2010	0.56	0.75
	Gowgaia Slope	2017	0.97	0.97
		2018	0.71	0.96
	Ramsay Island	2018	1	0.75
Sperm whale	SG̱ang Gwaay	2009	NA	NA
		2010	NA	NA
	Gowgaia Slope	2017	0.81	0.43
		2018	0.82	0.34
Porpoises	Gowgaia Shelf	2017	0.98	0.97
		2018	0.98	0.97
Delphinid clicks	Gowgaia Slope	2017	0.91	0.91
		2018	0.89	0.86
	Ramsay Island	2018	1	0.79
Delphnid whistles	Gowgaia Slope	2017	1	0.33
		2018	1	0.14
	Ramsay Island	2018	0.95	0.90
Baird's beaked whales	Gowgaia Slope	2017	0.95	0.83
		2018	0.48	0.70

NA: Not Applicable

2 Acoustic Detection ranges

2.1 Source Level Estimation

Each whale species produces vocalizations in different frequency bands and at different source levels. Table S4 lists the vocalization types, source level values, and frequency bands for each species of interest for this study based on a review of the scientific literature. The source levels of each species were represented in the Monte Carlo simulation by sampling respective Gaussian distributions with the means and standard deviations defined in Table S4 (see also Figure S1).

Table S4. Source levels and frequency bands of the vocalizations used in the Monte Carlo simulation to estimate the detection range of the species of interest.

Species	Vocalization type	Source level* (dB re 1 μPa)	Minimum frequency (Hz)	Maximum frequency (Hz)	Width of detection frequency bands (Hz)	Reference
Killer whales (northern residents)	Stereotyped pulsed vocalizations	152.6 ±5.9	1000	7900	300	[41]
Fin whales	20 Hz pulse	189 ± 5.8	15	30	15	[42]
Blue whales	B song-note	186 ± 2**	10	110	10	[26]
Humpback whales	Songs (unit E-whale L2)	160 ±4	50	9050	300	[43]
Gray whales	Moans (M3)	156.9 ±11.4	50	150	50	[44]
Sperm whales	Clicks-off-axis (Alaska)	186 ± 0.9	2000	14000	2000	[45]
Pacific white-sided dolphins	Whistles	138 ±8†	3000	14100	300	[46]

* Broadband source levels.

** The standard deviation was not provided in the literature for that species. We arbitrarily used a standard deviation of 2 dB re 1 μPa to allow some variations of source levels in the Monte Carlo simulation.

† There is currently no literature reporting source levels of that species. Source levels for bottlenose dolphin and Atlantic spotted dolphin whistles were used as surrogates.

The intensity of a vocalization is not always uniformly distributed across frequency bands; some frequency bands may have higher intensity than others. When available, this type of information is important to consider for estimating the vocalization detection range because different frequencies are attenuated and masked differently in the ocean. Unfortunately, this information is rarely found in the scientific literature, and most often only broadband source levels are reported. For this study, we derived energy distribution information for killer whale and blue whale vocalizations. Based on measurements performed at DFO's Whale Tracking Network hydrophone at Tilly Point, BC, the energy from killer whale pulsed vocalizations increases linearly at 3.7798 dB per 300 Hz band from 1000 to 1900 Hz and decreases at −1.12832 dB per 300 Hz band higher than 1900 Hz [47]. The source levels of killer whale vocalizations in the Monte Carlo simulation followed this frequency distribution. For the blue whale B song-note, McDonald, et al. [26] reported that the source level of the third harmonic (i.e., 50 Hz tone) is on average 10.3 dB lower than the fundamental (i.e., 16.5 Hz tone). Given that for blue whales the detection range modelling was performed in 10 Hz bands (Table S4), the broadband source level values of their vocalizations in the Monte Carlo simulation were distributed to have a slope of –2.575 dB per 10 Hz band. For all the other species, the source levels used in the Monte Carlo simulation were considered uniformly distributed across the frequency bands defined in Table S4.

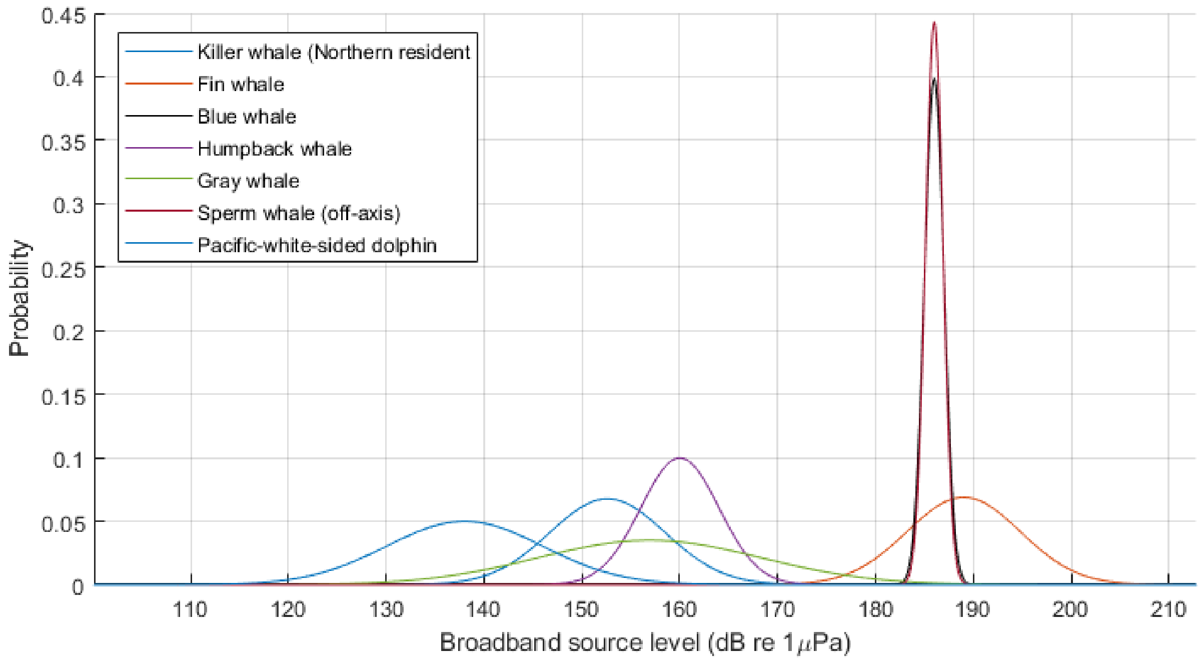

Figure S1. Gaussian probability distribution of source levels for each species used for the Monte Carlo simulation. The mean and standard deviation of each distribution were issued from the values in Table S4.

2.2 Vocalization Depth

Propagation loss is depth dependent, so the depth at which an animal vocalizes can greatly influence the maximum distance at which its vocalization can be detected. The Monte Carlo simulation randomly selected the vocalizing animal depth from a distribution covering the typical depth range for the whale's vocalization of interest. Depth models were defined by reviewing vocalization depth data from the scientific literature for each species of interest and representing these data using log-logistic, Gaussian, or Uniform distributions. When raw depth data were available, the parameters of the distributions were fitted using Maximum Likelihood. Otherwise, the distribution parameters were approximated using the information available from the literature. When the information was available, the model represented the depths at which the vocalizations are produced. If such information was unavailable, the model represented the depths at which the whales were distributed (but not necessarily vocalizing). For killer whales, the depth model was based on unpublished tag data collected by Brianna Wright from DFO and was used in a previous study for DFO by [47]. We did not find information in the literature about the depth distribution of white-sided dolphins, so we used the same model as killer whales for that species. Sperm whales were considered uniformly distributed from 10 to 500 metres (Figure S2).

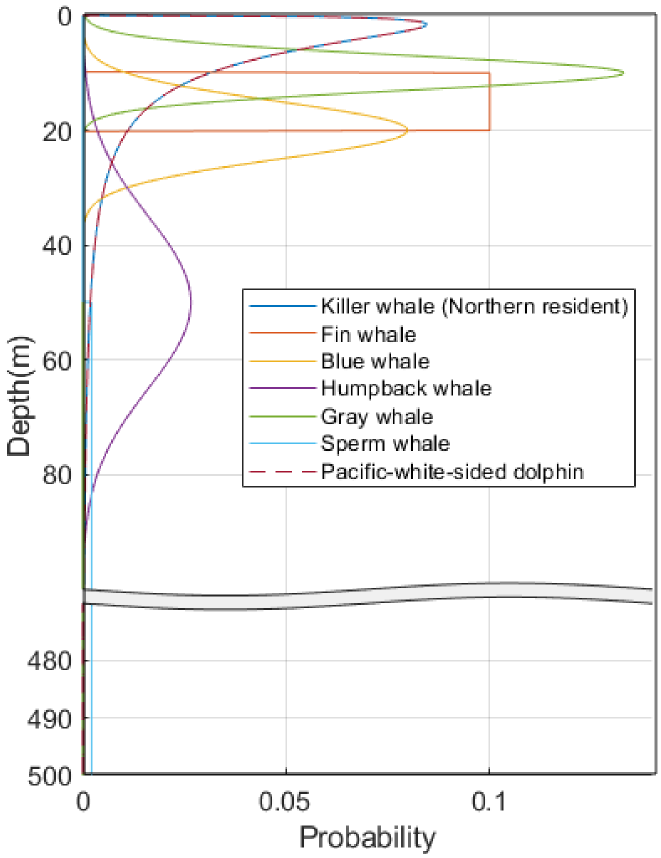

Figure S2. Depth models used in the Monte Carlo simulation for each species.

2.3 Propagation Loss Modelling

Propagation loss was modelled along four radials (Table S5) in different directions at Ramsay Island and Gowgaia Slope (Figure S3) to sample the propagation loss characteristics as a function of range and azimuth. The Bellhop Ray Tracing Model was used to calculate propagation loss for frequency bands with components above 1.0 kHz [48]. The model accounts for absorption in seawater, which can be important at the high frequencies considered in this study. Propagation loss for low frequencies (<1.0 kHz) was modelled with the Range-dependent Acoustic Model (RAM; [49]). Both models take as input the sound source location and depth, geoacoustic parameters of the ocean bottom, sound speed profiles for the water column, and a profile of the bathymetry along the modelled radials. Propagation losses were calculated at three frequencies within each 300 Hz band and then averaged to provide a propagation loss estimate for each frequency band of killer whales, humpback whales, sperm whales, and Pacific white-sided dolphins. In the frequency bands of fin, gray, and blue whales, propagation losses were calculated every 1 Hz.

All resulting propagation loss values were plotted as a function of range, and the data were fit with an equation of the form:

$$PL(f,z)=A(f,z)-n(f,z)\log R+\alpha(f,z)(R/1000)\ , \qquad (S1)$$

for frequency (f) in Hertz, depth (z) in metres, and range (R) in metres. The α(f,z) term in Equation S1 accounts for volume absorption in decibels per kilometer and is only used for curve fitting at the higher frequencies of killer whales, sperm whales, pacific white-sided dolphins, and part of the humpback whales. The resulting PL values were used in the Monte Carlo simulation for the corresponding depths and frequency bands.

Table S5. End point of the radials at each model location.

Location	Radial number	End point			
		Latitude (°)	Longitude (°)	Easting	Northing
Gowgaia Slope	1	49.58477	−128.15054	561400.53	5492811.41
	2	48.80029	−131.71308	300775.87	5408805.98
	3	51.63034	−137.41957	332542.06	5722698.33
	4	55.08089	−135.74876	452200.06	6104050.06
Ramsay Island	1	52.5921	−131.6670	319351.08	5830237.30
	2	53.1072	−129.8448	443445.87	5884529.10
	3	52.5238	−129.4891	466816.04	5819411.70
	4	51.7036	−128.2987	548461.27	5728305.30

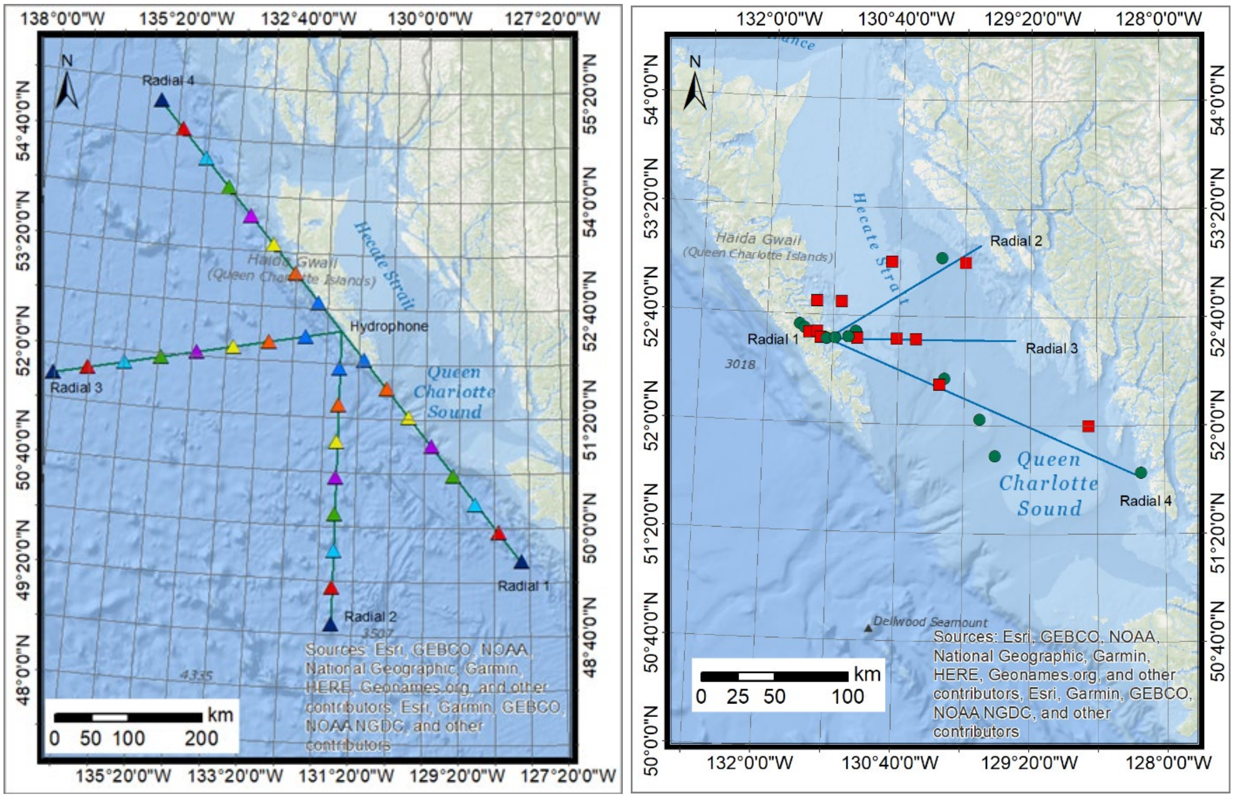

Figure S3. Location of the radials (green lines) used for estimating the detection range of whale and dolphin vocalizations at (left) Gowgaia Slope and (right) Ramsay Island. Map on the left: Coloured triangles show the locations of the sound speed profiles extracted from GDEM used for the propagation loss modelling. Red squares show the locations of the sound speed profiles extracted from GDEM used for the propagation loss modelling; green circles show the locations of the sound speed profiles extracted from CTD. Maps were created using ArcGIS v. 10.3.1.

2.3.1 Bathymetry

Water depths throughout the modelled locations were obtained from the ETOPO2 database [50] with a 2-minute resolution. Bathymetry values were extracted and re-gridded onto a Universal Transverse Mercator (UTM) Zone 9 coordinate projection with a regular grid spacing of 10 m.

2.3.2 Geoacoustics

The geoacoustic parameters of sediments are compressional or P-wave speed, shear or S-wave speed, density, P-wave attenuation, and S-wave attenuation. These parameters were derived for use in a previous noise mapping study [51] from data from two sources:

- Geological Survey of Canada (GSC) surficial sediment point sampling data.
- BC Marine Ecological Classification (BCMEC) polygon data.

Geoacoustic models were derived from bottom classification based on grain size [52].

2.3.3 Sound Speed Profile

For the Gowgaia Slope location, no conductivity, temperature and salinity (CTD) measurements were available, so summer water column sound speed profiles were computed from salinity and temperature profiles obtained from the Generalized Digital Environment Model (GDEM) version 3.0 in August (summer). In summer, heating of the surface results in a surface thermocline leading to higher surface sound speeds and downward acoustic refraction. For the propagation loss modelling, each radial was characterized by eight sound speed profiles (one every 50 km).

At the Ramsay Island location, water column sound speed profiles for summer were computed from conductivity, temperature, and salinity (CTD) measurements from the study area in August 2018. Sound speed profiles were calculated using the Del Grosso equation reformulated by Wong and Zhu for the 1990 International Temperature Scale [53]. In summer, heating the surface results in a downward refracting sound profile.

2.4 Detection range results

The detection range probabilities at Gowgaia Slope for blue whale B-song notes, fin whale 20 Hz pulses, humpback whale songs, killer whale pulsed vocalizations, and sperm whale off-axis clicks are summarized in Table S6.

The detection range probabilities at Ramsay Island for gray whale moans, fin whale 20 Hz pulses, humpback whale songs, killer whale pulsed vocalizations, and for Pacific white-sided dolphin whistles are summarized in Table S7.

Table S6. Median detection range (in metres) along each radial of Gowgaia slope for Probability of Detection, *P*, of 0.1, 0.5, and 0.9.

**Species**	**Radia l**	**Summer detection ranges (m)**		
		P = 0.1	*P* = 0.5	*P* = 0.9
Blue whale	1	33,600	28,300	22,200
	2	54,500	42,800	33,600
	3	56,500	44,300	33,600
	4	58,500	49,200	41,300
Fin whale	1	24,600	22,200	18,000
	2	67,100	52,700	34,800
	3	85,600	62,700	38,600
	4	56,500	49,200	38,600
Humpba ck whale	1	21,400	8,400	5,900
	2	18,600	6,200	3,800
	3	17,400	5,400	3,100
	4	24,600	7,600	4,500
Killer whale	1	24,600	1,600	500
	2	12,300	1,200	300
	3	9,300	1,200	500
	4	11,100	1,000	300
Sperm whale	1	144000	80000	56000
	2	74000	43000	32000
	3	72000	40000	28000
	4	102000	57000	41000

Table S7. Median detection range (in metres) along each radial of Ramsay Island for Probability of Detection, *P*, of 0.1, 0.5, and 0.9.

Species	Radial	Summer detection ranges (m)		
		P = 0.1	*P* = 0.5	*P* = 0.9
Gray whale	1	21700	21700	21700
	2	116900	116900	39800
	3	129700	129700	51000
	4	225600	225600	52700
Fin whale	1	21700	21700	21700
	2	116900	116900	21400
	3	129700	129700	47500
	4	225600	225600	125200
Humpback whale	1	21700	17100	5150
	2	40100	15900	5300
	3	55500	12700	2600
	4	71800	15200	2800
Killer whale	1	11700	5200	1600
	2	28800	12000	3300
	3	8500	2700	500
	4	6800	800	50
Pacific white-sided dolphin	1	1300	1100	600
	2	2400	1600	900
	3	800	600	300
	4	500	400	200

3 Diel patterns

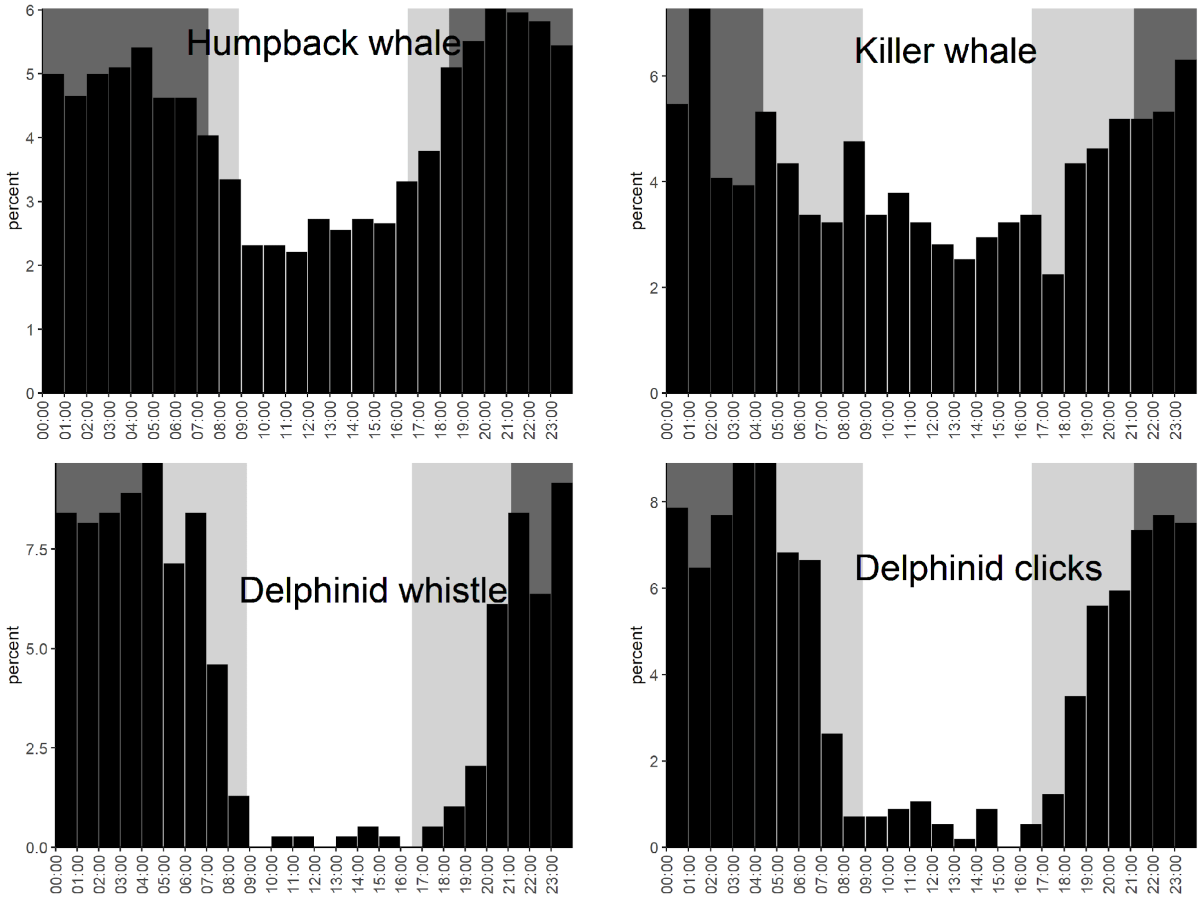

Figure S4. A) Diel pattern of humpback calls combined across all recording stations during winter months (November 1 to February 28). B) Diel pattern of killer whale calls combined across all recording stations and all recording periods. C) Diel pattern of delphinid whistles recorded at Ramsay Island. D) Diel pattern of delphinid clicks recorded at Ramsay Island. Vertical bars represent the percentage of time bins that have calls present in each 1 h time bin. Horizontal bars indicate periods of daylight (white), periods of darkness (dark gray), and periods of daylight or darkness depending on the time of recording (light gray).

4 Comparison of acoustic and visual cetaceans detections

Table S8. Summary of the number of confirmed and likely sightings of cetacean groups from 2009 and 2010 visual surveys in the area of the SG̱ang Gwaay hydrophone, and cetacean acoustic presence over the same time period in 2010. Groups of cetaceans vary in size and these numbers do not represent estimates of abundance

Cetacean species	SG̱ang Gwaay Visual Surveys July/August 2009 & July 2010	SG̱ang Gwaay PAM* July/August 2010
Fin whale	17 confirmed; 4 likely	Present
Humpback whale	43 confirmed; 8 likely	Present
Sei whale	1 likely	None detected
Sperm whale	14 confirmed	Present
Baird's beaked whale	3 confirmed	Outside recorder frequency range
Killer whale	2 confirmed	Present
Dall's porpoise	35 confirmed	Outside recorder frequency range
Pacific white-side dolphin	3 confirmed	Outside recorder frequency range

*Note the 2009 recordings started in September, and 2010 recordings overlapped only three days of visual surveys (15-17 July 2010); thus, 2009 and 2010 visual surveys were included assuming that the species acoustically detected in 2010 might also be recorded in 2009 if the recording period had started in July.

Table S9. Summary of the number of confirmed and likely cetacean sightings from 2018 visual surveys and cetacean acoustic presence in the area of the Ramsay Island hydrophone in the months immediately following the visual survey.

Cetacean species	Ramsay Island Visual Survey August 2018	Ramsay Island PAM* August 2018
Fin whale	6 confirmed	Present (Aug)
Humpback whale	9 confirmed	Present (Oct)
Minke whale	7 confirmed	None detected
Killer whale	2 confirmed	Present (Sep)
Dall's porpoise	8 confirmed	Outside recorder frequency range
Pacific white-side dolphin	1 confirmed	Potentially part of delphinid clicks
Risso's dolphin	1 confirmed	Potentially part of delphinid clicks

*Note the 2018 hydrophone did not get deployed until after the visual surveys were completed.

Table S10. Summary of the number of confirmed and likely cetacean sightings from 2018 visual surveys and cetacean acoustic presence in the area of the GS hydrophone over the same time period in 2018.

Cetacean species	Gowgaia Slope Visual Survey July 2018	Gowgaia Slope PAM July 2018
Blue whale	None observed	Present
Fin whale	9 confirmed	Present
Humpback whale	49 confirmed	Present
Sperm whale	2 confirmed	Present
Baird's beaked whale	None observed	Present (late June)
Killer whale	None observed	Present
Dall's porpoise	1 confirmed	Potentially part of porpoise clicks
Pacific white-side dolphin	1 confirmed	Potentially part of delphinid clicks

Table S11. Summary of the number of confirmed and likely cetacean sightings from visual surveys and cetacean acoustic presence in the area of the Gowgaia Slope hydrophone over the same time period in 2019.

Cetacean species or group	Gowgaia Slope Visual Surveys July 2019	Gowgaia Slope PAM July 2019
Fin whale	7 confirmed	None detected
Humpback whale	35 confirmed	Present
Sperm whale	2 confirmed	Present
Baird's beaked whale	None observed	Present
Killer whale	None observed	Present
Dall's porpoise	1 confirmed	Potentially part of porpoise clicks
Pacific white-side dolphin	None observed	Potentially part of delphinid clicks

*Note the hydrophone stopped recording early July at the same time that the visual survey was completed.

5 Environmental parameters

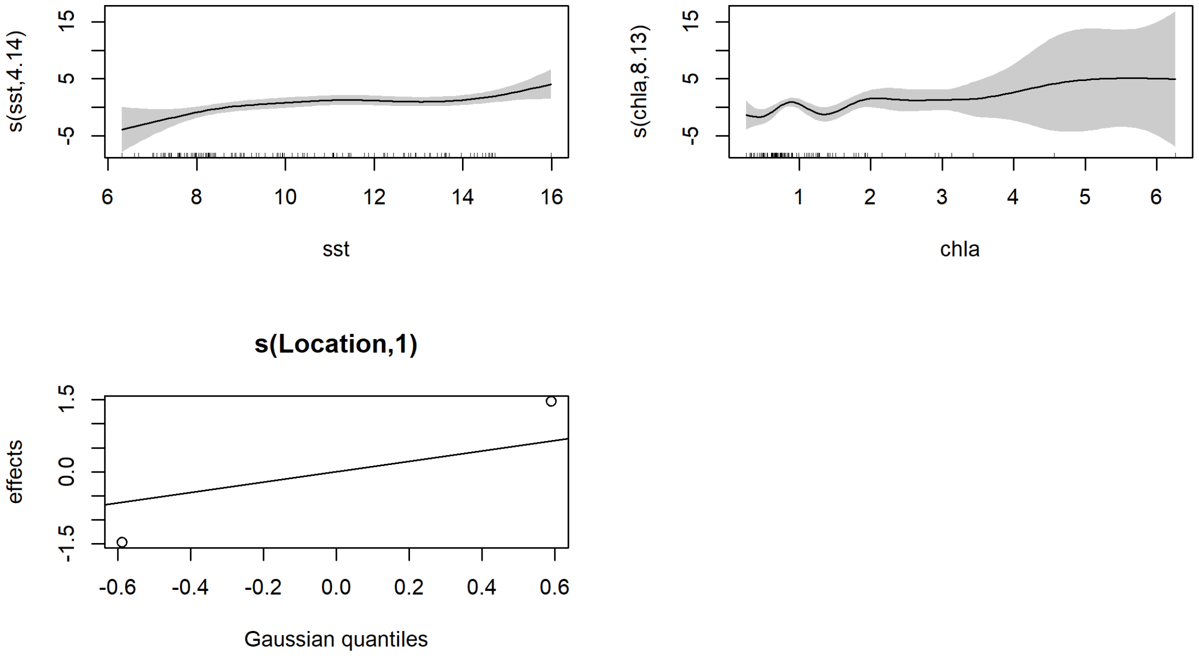

Figure S5. Results of the GAMM analysis for the correlation between sea surface temperature (SST), chlorophyll a (chla) and acoustic recorder locations and blue whale acoustic presence.

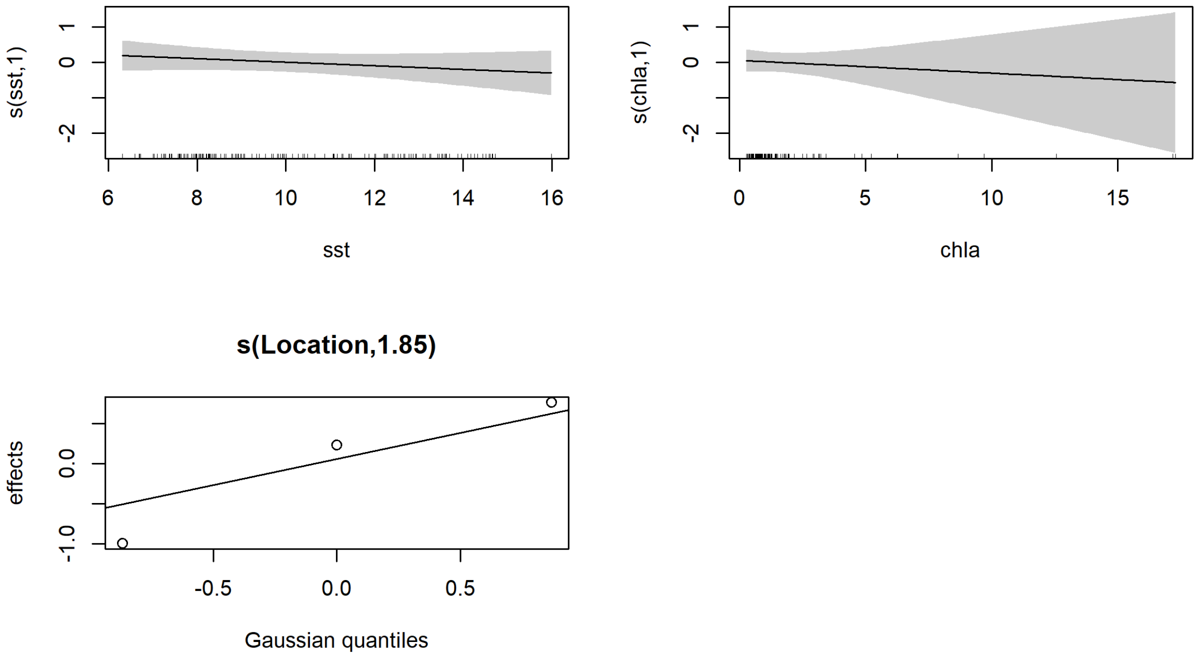

Figure S6. Results of the GAMM analysis for the correlation between sea surface temperature (SST), chlorophyll a (chla) and acoustic recorder locations and fin whale acoustic presence.

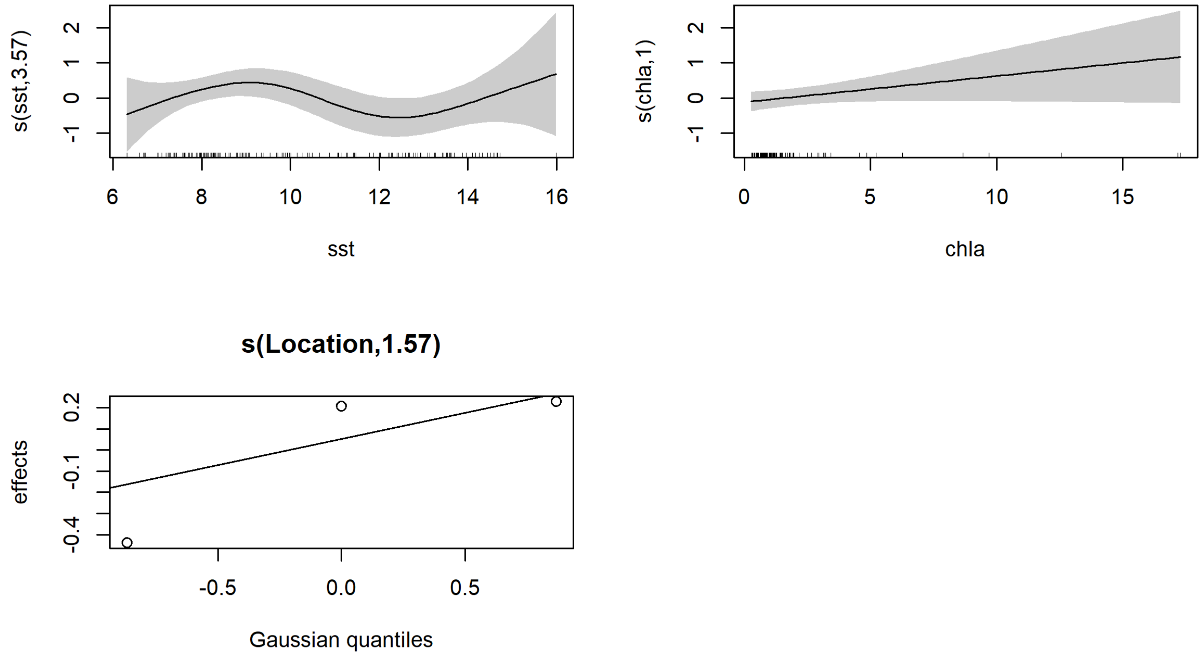

Figure S7. Results of the GAMM analysis for the correlation between sea surface temperature (SST), chlorophyll a (chla) and acoustic recorder locations and humpback whale acoustic presence.

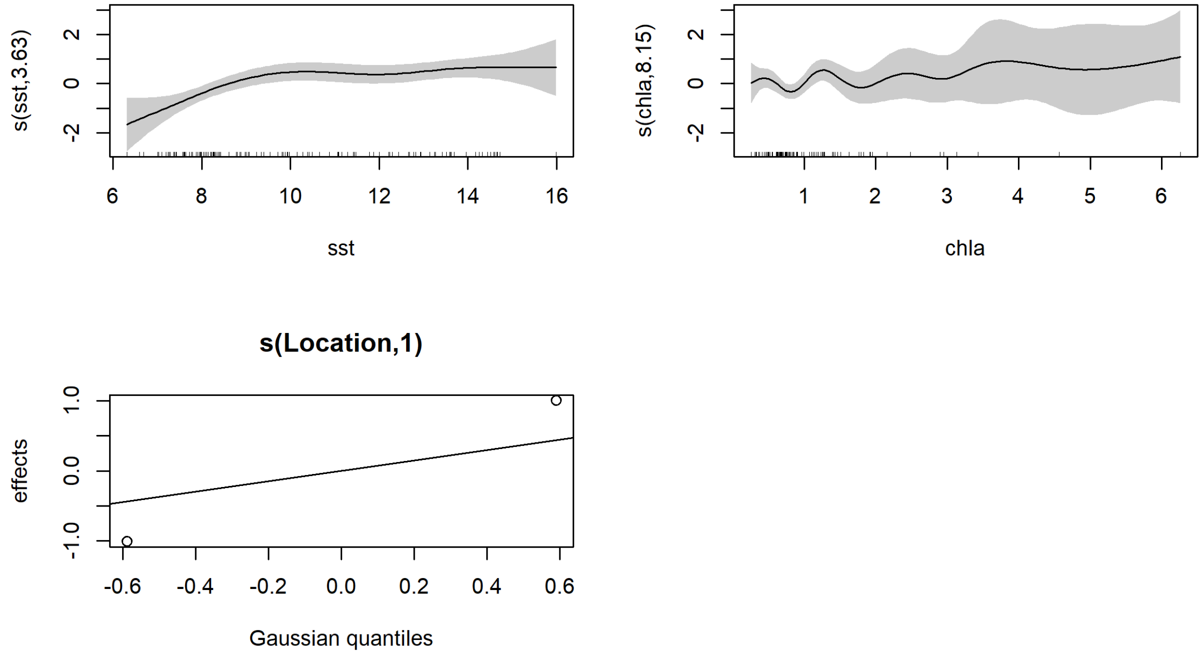

Figure S8. Results of the GAMM analysis for the correlation between sea surface temperature (SST), chlorophyll a (chla) and acoustic recorder locations and sperm whale acoustic presence.

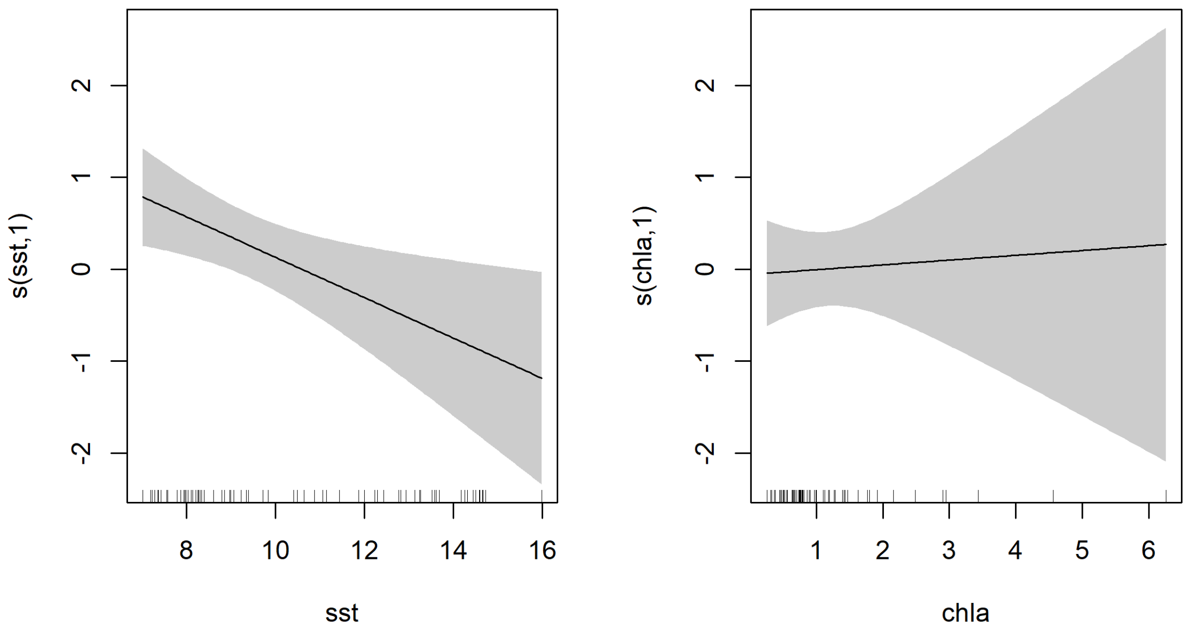

Figure S9. Results of the GAMM analysis for the correlation between sea surface temperature (SST) and chlorophyll a (chla) and Baird’s beaked whale acoustic presence.

References

1 Oleson, E. M. *et al.* Behavioral context of call production by eastern North Pacific blue whales. *Marine Ecology progress series* **330**, 269-284 (2007).

2 Watkins, W. A. Activities and underwater sounds of fin whales. *Scientific Reports of the Whales Research Institute* **33**, 83-117 (1981).

3 Baumgartner, M. F. *et al.* Low frequency vocalizations attributed to sei whales (*Balaenoptera borealis*). *Journal of the Acoustical Society of America* **124**, 1339-1349, doi:10.1121/1.2945155 (2008).

4 Rankin, S. & Barlow, J. P. Source of the North Pacific "boing" sound attributed to minke whales. *Journal of the Acoustical Society of America* **118**, 3346-3351 (2005).

5 Payne, R. S. & McVay, S. Songs of humpback whales. *Science* **173**, 585-597 (1971).

6 Rekdahl, M. L. *et al.* Non-song social call bouts of migrating humpback whales. *Journal of the Acoustical Society of America* **137**, 3042-3053 (2015).

7 McDonald, M. A. & Moore, S. E. Calls recorded from North Pacific right whales (*Eubalaena japonica*) in the eastern Bering Sea. *Journal of Cetacean Research and Management* **4**, 261-266 (2002).

8 Burnham, R., Duffus, D. & Mouy, X. Gray Whale (*Eschrictius robustus*) Call Types Recorded During Migration off the West Coast of Vancouver Island. *Frontiers in Marine Science* **5** (2018).

9 Au, W. W. L., Ford, J. K. B., Horne, J. K. & Allman Newman, K. A. Echolocation signals of free-ranging killer whales (*Orcinus orca*) and modeling of foraging for chinook salmon (*Oncorhynchus tshawytscha*). *Journal of the Acoustical Society of America* **115**, 901-909 (2004).

10 Ford, J. K. B. Acoustic behaviour of resident killer whales (*Orcinus orca*) off Vancouver Island, British Columbia. *Canadian Journal of Zoology* **67**, 727-745 (1989).

11 Møhl, B., Wahlberg, M., Madsen, P. T., Heerfordt, A. & Lund, A. The monopulsed nature of sperm whale clicks. *Journal of the Acoustical Society of America* **114**, 1143-1154, doi:10.1121/1.1586258 (2003).

12 Henderson, E. E., Hildebrand, J. A., Smith, M. H. & Falcone, E. A. The behavioral context of common dolphin (*Delphinus sp.*) vocalizations. *marine Mammal Science* **28**, 439-460, doi:10.1111/j.1748-7692.2011.00498.x (2012).

13 Soldevilla, M. S. *et al.* Classification of Risso's and Pacific white-sided dolphins using spectral properties of echolocation clicks. *The Journal of the Acoustical Society of America* **124**, 609-624, doi:10.1121/1.2932059 (2008).

14 Rankin, S., Oswald, J., Barlow, J. P. & Lammers, M. Patterned burst-pulse vocalizations of the northern right whale dolphin, *Lissodelphis borealis*. *Journal of the Acoustical Society of America* **121**, 1213-1218, doi:10.1121/1.2404919 (2007).

15 Baumann-Pickering, S. *et al.* False killer whale and short-finned pilot whale acoustic identification. *Endangered Species Research* **28**, 97-108 (2015).

16 Pérez, J. M., Jensen, F. H., Rojano-Doñate, L. & Aguilar de Soto, N. Different modes of acoustic communication in deep-diving short-finned pilot whales (*Globicephala macrorhynchus*). *Marine Mammal Science* **33**, 59-79, doi:10.1111/mms.12344 (2017).

17 Scheer, M., Hofmann, B. & Behr, I. P. Vocalizations of free-ranging short-finned pilot whales (*Globicephala macrorhynchus*) off Tenerife: Signal repertoire and characteristics (Poster presented at the annual conference of the European Cetacean Society, Las Palmas de Gran Canaria, Spain, 10-13 Mar 2003).

18 Baumann-Pickering, S. *et al.* Species-specific beaked whale echolocation signals. *Journal of the Acoustical Society of America* **134**, 2293-2301 (2013).

19 Stimpert, A. K. *et al.* Acoustic and foraging behavior of a Baird's beaked whale, *Berardius bairdii*, exposed to simulated sonar. *Scientific Reports* **4**, doi:10.1038/srep07031 (2014).
20 Zimmer, W. M. X., Johnson, M. P., Madsen, P. T. & Tyack, P. L. Echolocation clicks of free-ranging Cuvier's beaked whales (*Ziphius cavirostris*). *Journal of the Acoustical Society of America* **117**, 3919-3927 (2005).
21 Baumann-Pickering, S. *et al.* Spatio-temporal patterns of beaked whale echolocation signals in the North Pacific. *PLOS One* **9** (2014).
22 Merkens, K. *et al.* Clicks of dwarf sperm whales (*Kogia sima*). *Marine Mammal Science* **34**, 963-978 (2018).
23 Kyhn, L. A. *et al.* Clicking in a killer whale habitat: Narrow-band, high-frequency biosonar cliks of harbour porpoise (*Phocoena phocoena*) and Dall's porpoise (*Phocoenoides dalli*). *PLOS One* **8**, e63763 (2013).
24 Villadsgaard, A., Wahlberg, M. & Tougaard, J. Echolocation signals of wild harbour porpoises, *Phocoena phocoena*. *Journal of Experimental Biology* **210**, 56-64 (2007).
25 Lewis, L. A. *et al.* Context-dependent variability in blue whale acoustic behaviour. *Royal Society open science* **5**, 180241 (2018).
26 McDonald, M. A., Calambokidis, J., Teranishi, A. M. & Hildebrand, J. A. The acoustic calls of blue whales off California with gender data. *The Journal of the Acoustical Society of America* **109**, 1728-1735 (2001).
27 Clark, C. W., Borsani, J. & Notarbartolo-Di-sciara, G. Vocal activity of fin whales, *Balaenoptera physalus*, in the Ligurian Sea. *Marine Mammal Science* **18**, 286-295 (2002).
28 McDonald, M. A. & Fox, C. G. Passive acoustic methods applied to fin whale population density estimation. *The Journal of the Acoustical Society of America* **105**, 2643-2651 (1999).
29 Winn, H. E. & Winn, L. K. The song of the humpback whale *Megaptera novaeangliae* in the West Indies. *Marine Biology* **47**, 97-114, doi:10.1007/BF00395631 (1978).
30 Dunlop, R. A., Noad, M. J., Cato, D. H. & Stokes, D. The social vocalization repertoire of east Australian migrating humpback whales (*Megaptera novaeangliae*). *Journal of the Acoustical Society of America* **122**, 2893-2905 (2007).
31 Hendricks, B. *et al.* Automated localization of whales in coastal fjords. *The Journal of the Acoustical Society of America* **146**, 4672-4686 (2019).
32 Crane, N. L. & Lashkari, K. Sound production of gray whales, *Eschrichtius robustus*, along their migration route: A new approach to signal analysis. *Journal of the Acoustical Society of America* **100**, 1878-1886 (1996).
33 Rankin, S. & Barlow, J. Vocalizations of the sei whale *Balaenoptera borealis* off the Hawaiian Islands. *Bioacoustics* **16**, 137-145 (2007).
34 Crance, J. L., Berchok, C. L. & Keating, J. L. Gunshot call production by the North Pacific right whale *Eubalaena japonica* in the southeastern Bering Sea. *Endangered Species Research* **34**, 251-267 (2017).
35 Miller, P. J. Mixed-directionality of killer whale stereotyped calls: a direction of movement cue? *Behavioral Ecology and Sociobiology* **52**, 262-270 (2002).
36 Mouy, X., Bahoura, M. & Simard, Y. Automatic recognition of fin and blue whale calls for real-time monitoring in the St. Lawrence. *The Journal of the Acoustical Society of America* **126**, 2918-2928 (2009).
37 Mouy, X. *et al.* Automatic marine mammal monitoring off British-Columbia, Canada in *7th International DCLDE Workshop.* 13-16.
38 Moloney, J., Hillis, C., Mouy, X., Urazghildiiev, I. & Dakin, T. Autonomous multichannel acoustic recorders on the VENUS ocean observatory in *2014 Oceans-St. John's.* 1-6 (IEEE).

39 Martin, B., Kowarski, K., Mouy, X. & Moors-Murphy, H. Recording and identification of marine mammal vocalizations on the scotian shelf and slope in *2014 Oceans-St. John's.* 1-6 (IEEE).
40 Kowarski, K. A., Delarue, J. J.-Y., Gaudet, B. J. & Martin, S. B. Automatic data selection for validation: A method to determine cetacean occurrence in large acoustic data sets. *JASA Express Letters* **1**, 051201 (2021).
41 Miller, P. J. O. Diversity in sound pressure levels and estimated active space of resident killer whale vocalizations. *Journal of Comparative Physiology A* **192**, 449-459 (2006).
42 Weirathmueller, M. J., Wilcock, W. S. D. & Soule, D. C. Source levels of fin whale 20 Hz pulses measured in the Northeast Pacific Ocean. *The Journal of Acoustical Society of America* **133**, 741-749 (2013).
43 Au, W. W. L. *et al.* Acoustic properties of humpback whale songs. *Journal of the Acoustical Society of America* **120**, 1103-1110, doi:10.1121/1.2211547 (2006).
44 Guazzo, R. A. *et al.* Migratory behavior of eastern North Pacific gray whales tracked using a hydrophone array. *PLOS One* **12** (2017).
45 Mathias, D., Thode, A. M., Straley, J. & Andrews, R. D. Acoustic tracking of sperm whales in the Gulf of Alaska using a two-element vertical array and tags. *Journal of the Acoustical Society of America* **134**, 2446-2461 (2013).
46 Frankel, A. S., Zeddies, D. G., Simard, P. & Mann, D. Whistle source levels of free-ranging bottlenose dolphins and Atlantic spotted dolphins in the Gulf of Mexico. *Journal of the Acoustical Society of America* **135**, 1624-1631, doi:10.1121/1.4863304 (2014).
47 Austin, M. E. & Mouy, X. Modelling Acoustic Detection Ranges of Southern Resident Killer Whales. Document 01578, Version 1.0. Technical report by JASCO Applied Sciences for Fisheries and Oceans Canada. (2018).
48 Porter, M. B. & Bucker, H. P. Gaussian beam tracing for computing ocean acoustic fields. *Journal of the Acoustical Society of America* **82**, 1349-1359 (1987).
49 Collins, M. D. A split-step Padé solution for the parabolic equation method. *Journal of the Acoustical Society of America* **93**, 1736-1742 (1993).
50 NDCP [National Climatic Data Center]. 2-minute Gridded Global Relief Data (ETOPO2) v2. National Geophysical Data Center, NOAA. https://dx.doi.org/10.7289/V5J1012Q (Accessed 12 Sep 2019). (2006).
51 Erbe, C., MacGillivray, A. O. & Williams, R. Mapping Ocean Noise: Modelling Cumulative Acoustic Energy from Shipping in British Columbia to Inform Marine Spatial Planning. Report R2012-10. Report by Curtin University, JASCO Applied Sciences, and Sea Mammal Research Unit, Scottish Oceans Institute for World Wildlife Fund Canada. p 42., (2012).
52 Buckingham, M. J. Compressional and shear wave properties of marine sediments: Comparisons between theory and data. *Journal of the Acoustical Society of America* **117**, 137-152 (2005).
53 Wong, G. S. K. & Zhu, S. M. Speed of sound in seawater as a function of salinity, temperature, and pressure. *Journal of the Acoustical Society of America* **97**, 1732-1736 (1995).
